# Immunotherapy in Thyroid Cancer: Current Strategies and Challenges

**DOI:** 10.1002/cam4.71742

**Published:** 2026-04-16

**Authors:** Qian Zhang, Fan Wang, Wenbin Ma, Nan Liu, Yihan Lu, Ping Zhang

**Affiliations:** ^1^ Second Hospital of Dalian Medical University Dalian China; ^2^ Department of Endocrinology and Metabolism Second Hospital of Dalian Medical University Dalian China

**Keywords:** adoptive cell therapy, combination therapy, immune checkpoints, immunotherapy, PD‐1, PD‐L1, personalized treatment, targeted therapy, thyroid cancer, tumor microenvironment

## Abstract

Thyroid cancer is the most common endocrine malignancy, with an increasing global incidence. Despite advances in conventional treatments, such as surgery, radioactive iodine therapy, and TSH suppression, the management of advanced and aggressive forms, such as anaplastic thyroid carcinoma and refractory differentiated thyroid carcinoma, remains a challenge. Immunotherapy has emerged as a promising approach, with immune checkpoint inhibitors (ICIs) like PD‐1/PD‐L1 and B7‐H3 showing potential in enhancing antitumor immunity. Additionally, immune cells such as CD4+, CD8+, and M2 macrophages are key players in the tumor microenvironment, influencing treatment responses and serving as prognostic biomarkers. Combining ICIs with targeted therapies or adoptive cell therapies is being explored to overcome resistance and improve efficacy. However, challenges such as tumor heterogeneity and immune evasion mechanisms persist.

**Abbreviations**
AEs: adverse events
ATC: anaplastic thyroid carcinoma
CAR‐T: chimeric antigen receptor T‐cell
CTLs: cytotoxic T cells
DPP4: dipeptidyl peptidase 4
DTC: differentiated thyroid carcinoma
FTC: follicular thyroid carcinoma
HT: Hashimoto's thyroiditis
I^131^
: radioactive iodine‐131
ICIs: immune checkpoint inhibitors
IET: iodine‐induced thyroiditis
irAEs: immune‐related adverse events
lncRNAs: long non‐coding RNAs
m6A: N6‐methyladenosine
MTC: medullary thyroid carcinoma
NK: natural killer
ORR: objective response rate
OS: overall survival
OTC: oncocytic thyroid cancer
PDTC: poorly differentiated thyroid carcinoma
PTC: papillary thyroid carcinoma
RAIR: radioiodine refractory
SIGLECs: sialic acid‐binding immunoglobulin‐type lectin
TAMs: tumor‐associated macrophages
TC: thyroid Cancer
T cells: T lymphocytes
TCR: T‐cell receptor
TILs: tumor‐infiltrating lymphocytes
TIM‐3: T‐cell immunoglobulin and mucin‐domain containing protein‐3
TKIs: tyrosine kinase inhibitors
TME: tumor microenvironment
Tregs: regulatory T cells
TSH: thyroid stimulating hormone

## 1 Introduction

Thyroid cancer (TC) is the most prevalent endocrine malignancy [1], and its incidence has been steadily rising worldwide in recent decades. Over the past three decades, the global incidence of TC has increased by nearly 87.5% [2], driven by factors such as excessive iodine intake, overdiagnosis, exposure to ionizing radiation, environmental endocrine disruptors, and a personal or family history of thyroid disease [3]. The increasing incidence and associated morbidity of TC place a substantial burden on healthcare systems and society.

TC can be broadly classified into differentiated thyroid carcinoma (DTC), anaplastic thyroid carcinoma (ATC), poorly differentiated thyroid carcinoma (PDTC), and medullary thyroid carcinoma (MTC). DTC is the most common subtype, comprising both papillary thyroid carcinoma (PTC)—which is typically associated with BRAF/RAS‐like mutations—and follicular thyroid carcinoma (FTC), both of which arise from thyroid follicular cells. ATC is an aggressive and poorly prognostic form of TC with a rare incidence, while PDTC represents an intermediate subtype, exhibiting more aggressive behavior than DTC but a better prognosis than ATC. MTC remains a distinct subtype, originating from parafollicular C cells, as defined in the 2022 WHO Classification of Endocrine and Neuroendocrine Tumors [4].

Current treatment strategies for TC primarily include total or near‐total thyroidectomy, radioactive iodine‐131 (I^131^) therapy and thyroid stimulating hormone (TSH) suppression. However, these therapies have their limitations: surgical treatment carries inherent risks and potential postoperative complications; I^131^ therapy can impact patients' long‐term health, including an increased risk of second primary cancers [5]; and TSH suppression therapy requires long‐term medication, which may increase fracture risk and cardiovascular issues, particularly in older patients [5]. More critically, these treatments have limited efficacy in the management of recurrent or metastatic DTC and in more aggressive forms such as PDTC and ATC [6].

In response, new treatment modalities, including targeted therapies and immunotherapy, have emerged as promising options for TC management and are already being applied in the treatment of various cancers. Unlike conventional therapies, immunotherapy harnesses the body's immune system to identify and destroy cancer cells. Recent advancements in understanding the immunological landscape of TC have paved the way for the development of novel immunotherapeutic approaches. These strategies hold significant potential to improve outcomes in TC, especially in cases resistant to traditional treatments. The growing emphasis on personalized immunotherapy offers hope for more effective and targeted treatments for TC patients [7]. This review aims to provide a comprehensive update on the latest immunological mechanisms and clinical trial developments in TC, highlighting the transformative potential of immunotherapy in managing this complex malignancy.

## 2 Tumor‐Associated Immune Cell Characteristics

**FIGURE 1:**
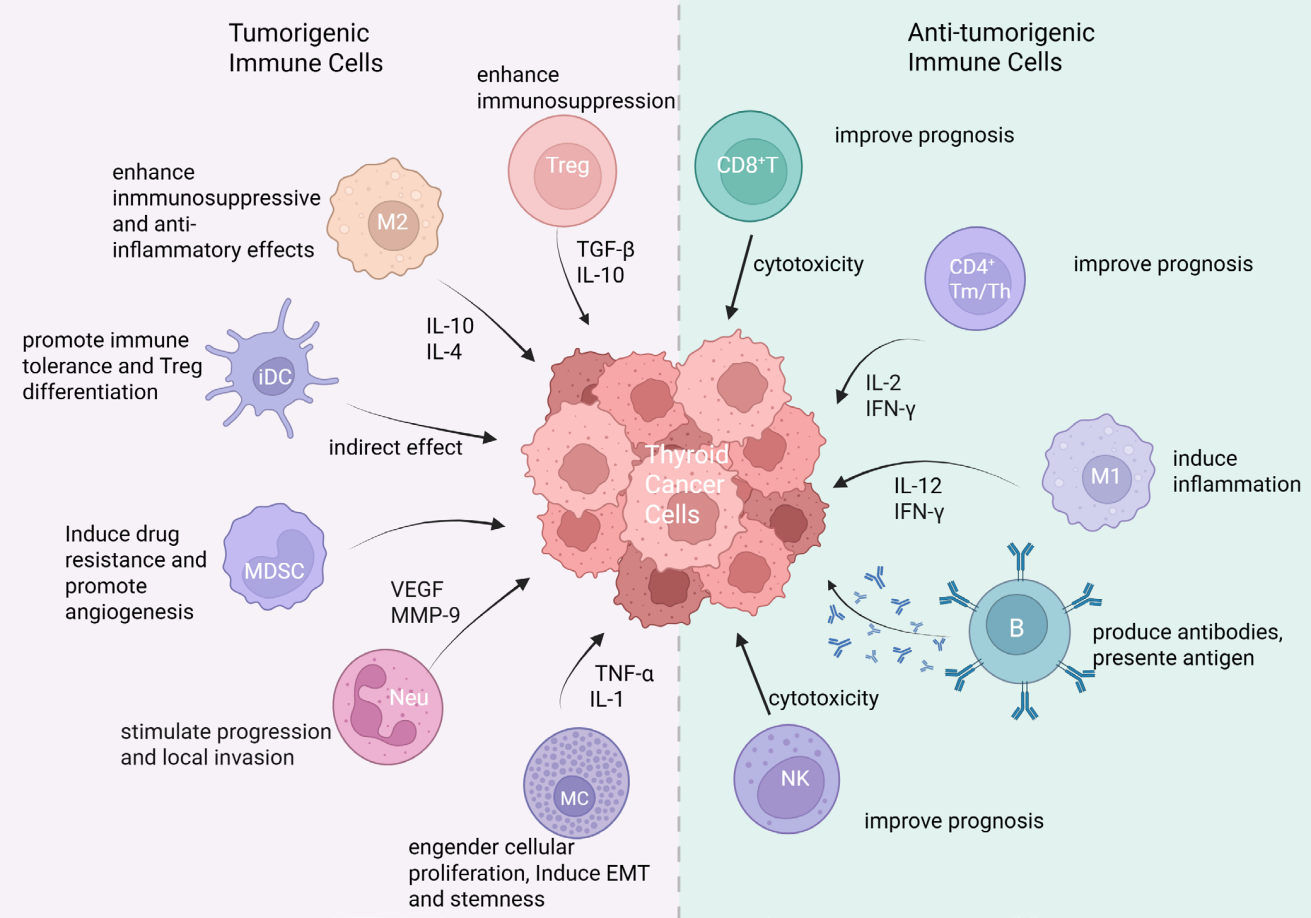
Immune landscape of thyroid cancer: Tumor‐promoting vs. tumor‐suppressing cells. B, B cells; CD4+ Tm/Th, CD4+ memory T cells/helper T cells; CD8+, CD8+ T cells; EMT, epithelial‐mesenchymal transition; iDC, immature dendritic cells; M1, M1 macrophages; M2, M2 macrophages; MC, mast cells; MDSCs, myeloid‐derived suppressor cells; Neu, neutrophils; NK, natural killer cells; TC, thyroid cancer; Treg, regulatory T cells. (Figure was created with BioRender.com).

TC is infiltrated by a variety of adaptive and innate immune cells that exert both pro‐tumorigenic and anti‐tumorigenic effects. The concept of cancer immunoediting, proposed by Dunn and Schreiber in 2002, suggests that the immune system not only identifies and eliminates tumors but also aids in enabling tumor immune evasions [8]. Recent advances in immunotherapy have affirmed the potential for treating relapsed and refractory cancers. However, therapeutic efficacy remains limited, partly due to the cancer's ability to evade immune surveillance and adapt to immune pressure. Several mechanisms contribute to immune evasion in cancer, including T lymphocyte (T cell) exhaustion, upregulation of inhibitory receptors such as PD‐1, cell‐mediated repression (e.g., by regulatory T cells [Tregs]), secretion of suppressive cytokines, nutrient depletion, metabolic dysfunction, immune escape, and immunosuppression within the tumor microenvironment (TME) [9]. As integral components of the TME, studies have shown that Tregs, neutrophils, dendritic cells, M2 macrophages, and resting mast cells play a tumorigenic role in the PTC microenvironment, while CD8+ T cells, CD4+ memory T cells, B cells, M1 macrophages, and NK cells portray a protective role [10, 11, 12] (Figure 1).

### 2.1 T Cells in TC

T cells are a class of lymphocyte that mature in the thymus and can be classified into helper T cells, cytotoxic T cells (CTLs), and Tregs. Studies have demonstrated that in PTC patients harboring BRAF mutations, the expression of tumor‐infiltrating lymphocytes (TILs), particularly CD4+ T cells and Tregs, is elevated [13]. Furthermore, Wang et al. [14] reported that in progressive PTC, CD8+ T cells exhibit increased levels of exhaustion compared to adjacent normal tissues. The co‐occurrence of these exhausted CD8+ T cells with elevated Treg levels suggests impaired immune surveillance. Another study found that, in comparison to multinodular goiter and peripheral blood samples, PTC tissues contain a higher abundance of Tregs, with recurrent patients showing an increased proportion of effector Tregs [15]. CXCL13+ T lymphocytes, enriched in ATC, may facilitate the formation of early tertiary lymphoid structures, playing a central role in ATC's response to immunotherapy [16]. γδ T cells also hold significant potential, as their infiltration inversely correlates with the differentiation status of TC [17]. One study proposed that the cell surface glycoprotein dipeptidyl peptidase 4 (DPP4) is positively correlated with the infiltration of various immune cells, including B cells, CD8+ T cells, neutrophils, macrophages, and dendritic cells [18]. Moreover, Jing et al. [19] discovered that inhibiting DPP4 alleviates CD8+ T cell exhaustion and reduces IL‐13 secretion, effectively disrupting the IL13‐IL13RA2 axis. This disruption promotes mesenchymal‐to‐epithelial transition in PTC cells, influencing both immune responses and tumor plasticity.

Current immunotherapy efforts largely rely on the targeted protection provided by tumor‐reactive T cells, primarily through immune checkpoint inhibitors (ICIs), such as PD‐1/PD‐L1 and CTL‐4, as well as adoptive cell therapies, such as chimeric antigen receptor (CAR) T cell therapy [9], which will be discussed in detail later in this review regarding their progress in TC.

### 2.2 Macrophages in TC

Macrophages are the most abundant cell population in the TME and are classified into M1, M2, and unstimulated M0 types based on their activation state and functional roles [20]. M1, classically activated macrophages, primarily produce pro‐inflammatory cytokines and exhibit anti‐tumor functions, whereas M2, alternatively activated macrophages, produce immunosuppressive cytokines that promote tumor progression [21]. Studies have shown that the infiltration of macrophages in PTC is significantly higher than in adjacent normal tissues or benign tumors [12, 22]. Tumor‐associated macrophages (TAMs), which often refer to both M1 and M2 types, contribute to tumor progression as both “immune suppressors” and “tumor promoters,” highlighting the importance of identifying macrophage‐specific targets to optimize current immunotherapies [23].

TAMs play a critical role in TC, especially in ATC, implicate to immune suppression [24, 25, 26], angiogenesis [16, 27], invasion [27] and therapy resistance [28]. In ATC, more than 50% of the infiltrating cells are TAMs, which are polarized into pro‐tumor M2 types by paracrine signals secreted by ATC cells [29]. ATC‐specific macrophage subpopulations, such as IL2RA+ VSIG4+ TAMs, co‐expressed M1 and M2 markers, are associated with BRAF and RAS signaling, as well as increased TILs like B cells, CD8+ T cells and Tregs. High infiltration of IL2RA+ VSIG4+ ATAMs correlates with a favorable patient prognosis [30]. The M2 macrophage subtype has garnered particular attention in the search for new therapeutic targets. In xenograft models of ATC, Palacios et al. [31] found that 24%–28% of CD45+ immune cells were macrophages, with 40% of these macrophages exhibiting an M2‐like phenotype. In human ATC, macrophage marker expression was positively correlated with T‐cell immunoglobulin and mucin‐domain containing protein‐3 (TIM‐3) expression, which may represent a newly identified immune checkpoint in macrophages. In addition to M1 and M2 macrophages, Li et al. [32] identified SPP1+ macrophages in TC with immunosuppressive functions, and CD14+ monocytes were found to contribute to tumor progression and angiogenesis. The SPP1‐CD44 and MIF‐CD74 axes, mediating communication between SPP1+ macrophages and T cells, could potentially reverse the immunosuppressive TME, enhancing the efficacy of immunotherapy.

### 2.3 Myeloid‐Derived Suppressor Cells in TC

Myeloid‐derived suppressor cells (MDSCs) are a heterogeneous population of immature myeloid cells [33], which have been identified to be one of the major contributors of immunosuppressive populations in TME [34]. Major subtypes include early, polymorphonuclear, and monocytic MDSCs, and although they differ in their phenotype and potency, they share a common ability to suppress effector T cell activity, similar to M2‐polarized TAMs [35]. Research has found that in the bone marrow, myeloid cells in TC patients undergo significant transcriptional and functional changes prior to tumor infiltration. These changes are already initiated in the bone marrow, suggesting that they play an active role in the formation of the TME [36]. Additionally, some scholars have proposed that BRAF V600E promotes the development of TC by facilitating the recruitment of MDSCs [37]. Studies have shown that the levels of circulating MDSCs detected in peripheral blood of TC patients are closely associated with tumor staging [35] and poor prognosis [38]. Specifically, for ATC, a study utilized multiplex immunofluorescence and immunohistochemistry techniques to retrospectively analyze surgical specimens from 26 ATC patients, proposing that the presence of MDSCs in the ATC TME may lead to resistance to anti‐PD therapy [39]. Additionally, long‐term survivors of ATC had lower tumor‐infiltrating MDSC counts, suggesting that MDSCs play a role in preparing the pre‐metastatic microenvironment before distant metastasis manifests clinically [40]. However, other studies have found that when mice tumors exhibit the greatest response to combination therapy with BRAF inhibitors and anti‐PD‐1/PD‐L1 antibodies, there is a significant reduction in the monocyte MDSC‐like cell component, but MDSC‐like cells remain low during tumor regeneration. This suggests that MDSCs may play a role in the response to combination therapy but not in resistance to combination therapy [41].

### 2.4 Other Immune Cells in TC

Beyond T cells, macrophages and MDSCs, other immune cells such as dendritic cells [14, 42], mast cells [43], natural killer (NK) cells, and eosinophils [44] also exhibit distinct patterns in TC. For instance, the infiltration of activated dendritic cells and M0 macrophages is increased in PTC compared to normal tissues, whereas the infiltration of activated NK cells and eosinophils is decreased. These patterns of immune cell infiltration are closely associated with the tumor's clinicopathological features and may influence patient prognosis [45]. Moreover, the cell surface glycoprotein DPP4, also known as CD26, positively correlates with the infiltration of B cells, CD8+ T cells, neutrophils, macrophages, and dendritic cells. Inhibiting DPP4 can alleviate CD8+ T cell exhaustion, impact immune responses, and influence tumor plasticity [46].

## 3 Immune Checkpoints in TC

In the TME, immune surveillance is often suppressed through two main mechanisms: signaling inhibition and metabolic suppression. Signaling inhibition involves tumor cells downregulating the activity of stimulatory immune receptors while upregulating the activity of inhibitory immune receptors. Over the past few decades, numerous inhibitory immune receptors have been identified and studied in cancer, including PD‐1, CTLA‐4, LAG3, TIM‐3, TIGIT, and BTLA. These receptors are collectively known as “immune checkpoints” [47]. Most immune checkpoint molecules are expressed on cells of the adaptive immune system as well as those of the innate immune system. However, the expression of immune checkpoint molecules on tumor cells plays a critical role in maintaining several malignant behaviors [48]. Blocking the activation of inhibitory immune receptors has been shown to re‐activate the anti‐tumor functions of immune cells. This concept has been experimentally validated and is now applied in the clinical treatment of various cancer types [49, 50]. By analyzing the expression of immune checkpoint molecules through tissue staining, researchers can gain deeper insights into the mechanisms of immune evasion in TC and identify patients who may benefit from immunotherapy.

### 3.1 PD‐1/PD‐L1 Axis

The PD‐1/PD‐L1 axis is one of the most studied immune checkpoint pathways. PD‐1 is typically expressed on TILs, while PD‐L1 is expressed on antigen‐presenting cells and tumor cells. The interaction between these immune checkpoint proteins plays a key role in regulating immune checkpoints, tumor immune evasion, and T‐cell function restoration, and serves as a useful but imperfect biomarker for predicting patient responses to anti‐PD‐1 or anti‐PD‐L1 antibody treatments across various tumor types [51]. PD‐L1 overexpression on tumor cells binds to PD‐1 on T cells, transmitting inhibitory signals that lead to T‐cell exhaustion (e.g., reduced proliferation and cytokine secretion), ultimately resulting in tumor immune evasion [52]. This interaction forms the biological basis of immune surveillance escape in tumors [53]. PD‐L1 can also modulate metabolic pathways (e.g., mTOR signaling) and participate in DNA damage response, regulating gene expression via intracellular signaling to promote tumor survival and progression [54, 55]. The expression of PD‐1/PD‐L1 is regulated by post‐translational modifications such as phosphorylation, ubiquitination (e.g., MDM2‐mediated PD‐1 degradation), and glycosylation [56, 57, 58], offering potential targets for developing new inhibitors. Interestingly, tumor‐targeted PEG‐PD1‐PDL1 fusion proteins can block the PD‐1/PD‐L1 interaction while reducing T‐cell exhaustion, achieving an 88.9% tumor suppression rate in animal models [59].

The expression of PD‐1/PD‐L1 in TC varies significantly across different cancer types and is influenced by factors such as sample size, antibody type, and positive threshold (Table 1). Over the past 5 years, the most research has been conducted on ATC, with positive rates ranging from 47% (7/15) [78] to 80% (8/10) [63]. Most large‐scale studies reported positive rates between 60%–80%, which is significantly higher than in other tumor types. For PDTC, reported PD‐L1 positivity rates are generally lower, with multiple teams reporting no PD‐L1 positivity in their tested PDTC samples [60, 61]. When the positivity threshold was set to tumor proportion score (TPS) ≥ 5%, the highest reported positivity rate was only 47% [68]. For FTC, reported PD‐L1 positivity rates ranged from 10% [66] to 59.72% [69]. For PTC, the reported PD‐L1 positivity rates vary significantly, with several studies of larger sample sizes reporting positivity rates around 33% [64, 70, 72]. Studies on PD‐L1 staining in MTC are also numerous, but the reported positive rates for MTC are lower, all below 20%.

**TABLE 1:** PD‐L1 positivity in different subtypes of TC.

Author	Subtypes of TC	Sample type	Number of samples	Antibody clone	Number of PD‐L1 positive samples	PD‐L1 positivity	Positivity threshold
ATC							
Piermattei et al. [60]	ATC	Thyroid biopsies or surgical samples	30	Clone 22C3	19	63.30%	TPS ≥ 1%
Shobab et al. [61]	ATC	Surgical samples	13	Clone SP142	Not mentioned.^a^	69.00%	Cells with membranous staining.^b^
Anand et al. [62]	ATC	Surgical samples	3	Clone SP263	3	100%	Cells with membranous staining.^b^
Sajedi Shacker et al. [63]	ATC	Surgical samples	10	Not mentioned	8	80.00%	Cells with membranous staining.^b^
Zhu et al. [64]	ATC	Surgical samples	33	Clone 22C3	22	66.67%	TPS ≥ 1%
Agarwal et al. [65]	ATC	Surgical samples	179	Clone SP263	131	73.18%	TPS ≥ 1%
Boruah et al. [66]	ATC	Surgical samples	15	Clone SP263	11	73.33%	TPS ≥ 1%
Luo et al. [67]	ATC	Surgical samples	22	E1L3N	14	63.60%	CPS ≥ 1
Adam et al. [68]	ATC	Thyroid biopsies or surgical samples	93	Clone 28–8	68	73%	TPS ≥ 5%
PDTC							
Piermattei et al. [60]	PDTC	Thyroid biopsies or surgical samples	35	Clone 22C3	0	0%	TPS ≥ 1%
Shobab et al. [61]	PDTC	Surgical samples	10	Clone SP142	Not mentioned.^a^	10%	Cells with membranous staining.^b^
Boruah et al. [66]	PDTC	Surgical samples	10	Clone SP263	1	10%	TPS ≥ 1%
Luo et al. [67]	PDTC	Surgical samples	44	E1L3N	14	31.80%	CPS ≥ 1
Adam et al. [68]	PDTC	Thyroid biopsies or surgical samples	47	Clone 28–8	22	47%	TPS ≥ 5%
FTC							
Sajedi Shacker et al. [63]	FTC	Surgical samples	21	Not mentioned	4	19.05%	Cells with membranous staining.^b^
Shobab et al. [61]	FTC	Surgical samples	20	Clone SP142	Not mentioned.^a^	11%	Cells with membranous staining.^b^
Anand et al. [62]	FTC	Surgical samples	19	Clone SP263	3	16%	Cells with membranous staining.^b^
Boruah et al. [66]	FTC	Surgical samples	10	Clone SP263	1	10%	TPS ≥ 1%
Lin et al. [69]	FTC	Surgical samples	72	Clone 2B11D11	43	59.72%	TPS ≥ 1%
PTC							
Sajedi Shacker et al. [63]	PTC	Surgical samples	48	Not mentioned	15	31.25%	Cells with membranous staining.^b^
Santana et al. [70]	PTC	Surgical samples	121	Clone SP263	40	33.06%	TPS ≥ 1%
Anand et al. [62]	PTC	Surgical samples	64	Clone SP263	21	33%	Cells with membranous staining.^b^
Bernadett et al. [71]	PTC	Surgical samples	89	Clone 22C3	64	71.90%	TPS ≥ 1%
Boruah et al. [66]	PTC	Surgical samples	30	Clone SP263	26	86.67%	TPS ≥ 1%
Luo et al. [67]	PTC	Surgical samples	168	E1L3N	17	10.10%	CPS ≥ 1
Adam et al. [68]	PTC	Thyroid biopsies or surgical samples	21	Clone 28–8	12	57%	TPS ≥ 5%
Siraj et al. [72]	PTC	Not mentioned	1458	E1L3N	473	32.40%	TPS ≥ 5%
Zhu et al. [64]	Tall cell PTC	Surgical samples	28	Clone 22C3	14	50.00%	TPS ≥ 1%
	Solid PTC	Surgical samples	20	Clone 22C3	9	45.00%	TPS ≥ 1%
	Diffuse sclerosing PTC	Surgical samples	31	Clone 22C3	5	16.13%	TPS ≥ 1%
	Columnar cell PTC	Surgical samples	15	Clone 22C3	1	6.67%	TPS ≥ 1%
	Classic PTC	Surgical samples	32	Clone 22C3	7	21.88%	TPS ≥ 1%
	Aggressive subtypes of PTC	Surgical samples	109	Clone 22C3	34	31.19%	TPS ≥ 1%
	Hobnail PTC	Surgical samples	15	Clone 22C3	5	33.33%	TPS ≥ 1%
Shobab et al. [61]	Classic PTC	Surgical samples	81	Clone SP142	Not mentioned.^a^	28.50%	Cells with membranous staining.^b^
	Papillary TC Follicular Variant	Surgical samples	30	Clone SP142	Not mentioned.^a^	0	Cells with membranous staining.^b^
Kovacevic et al. [73]	Papillary thyroid microcarcinoma (PTMC)	Surgical samples	99	Clone 22C3	61	61.60%	Allred score ≥ 3
MTC							
Sajedi Shacker et al. [63]	MTC	Surgical samples	21	Not mentioned	3	14.29%	Cells with membranous staining.^b^
Shobab et al. [61]	MTC	Surgical samples	11	Clone SP142	Not mentioned.^a^	0%	Cells with membranous staining.^b^
Wusiman et al. [74]	MTC	Surgical samples	190	Clone 22C3	13	6.84%	CPS ≥ 1
Bai et al. [75]	MTC	Surgical samples	49	Clone SP142	9	18.36%	CPS ≥ 1
	MTC	Surgical samples	49	Clone 22C3	6	12.24%	CPS ≥ 1
Other types							
Shobab et al. [61]	Oncocytic TC	Surgical samples	8	Clone SP142	Not mentioned.^a^	71%	Cells with membranous staining.^b^
Piermattei et al. [60]	High‐grade differentiated thyroid carcinomas (DHGTC)	Thyroid biopsies or surgical samples	8	Clone 22C3	5	62.50%	TPS ≥ 1%
Harahap et al. [76]	Aggressive histological types	Surgical samples	26	Clone 22C3	12	46.15%	TPS ≥ 1%
	Less aggressive histological types	Surgical samples	26	Clone 22C3	4	15.38%	TPS ≥ 1%
Zhu et al. [64]	DTC with distant metastasis	Surgical samples	21	Clone 22C3	6	28.57%	TPS ≥ 1%
	Differentiated high‐grade TC	Surgical samples	7	Clone 22C3	6	85.71%	TPS ≥ 1%
Do‐Youn Oh et al. [77]	PTC/FTC (failure of or intolerance to prior therapy)	/	103	Clone 22C3	46	44.67%	CPS ≥ 1

*Note:* Only studies with a total sample size greater than 50 in the past 5 years are shown.

Abbreviations: ATC, anaplastic thyroid carcinoma; CPS, combined positive score; FTC, follicular thyroid carcinoma; MTC, medullary thyroid carcinoma; PDTC, poorly differentiated thyroid carcinoma; PTC, papillary thyroid carcinoma; TC, thyroid carcinoma; TPS, tumor proportion score.

^a^ Data for PD‐L1 expression were available for part of the patients.

^b^ Only cells showing membranous staining were considered positive, or the staining was regarded as positive only when cells had membranous PD‐L1 expression.

The difference in PD‐L1 positivity rates is one of the factors that affect the efficacy of anti‐PD‐1/PD‐L1 immunotherapy. However, the efficacy among different TC types remains complex due to variations in subtypes and individual differences. There are many other possible causes of the development of PD‐1/PD‐L1 antibody resistance. For example, while PD‐L1 expression is relatively high in ATC, existing studies have shown that PD‐1 expression on CTLs in ATC is typically low or absent [39, 67, 79], and this “target‐missing” situation is detrimental to treatment efficacy [39]. In addition, TC typically has a low tumor mutational burden (TMB), such as in PTC and MTC [80, 81]. A low TMB implies fewer neoantigens produced by the tumor, and the absence of tumor‐specific antigens may hinder the effective activation of immune responses by anti‐PD‐1/PD‐L1 therapy [82, 83]. Additionally, changes or interactions among components of the TME [39, 84, 85, 86, 87], expression of other alternative immune checkpoints [88, 89], as well as abnormalities in cellular signaling [90, 91] or antigen processing and presentation [83, 92], which have been reported in other cancers [93], may also influence the efficacy of anti‐PD‐1/PD‐L1 immunotherapy in TC.

### 3.2 B7‐H3

B7‐H3, also known as CD276, is a member of the B7‐CD28 family and has both anti‐tumor and pro‐tumor effects [94]. It is rarely expressed in normal tissues but is highly expressed in several human cancers, including TC [95, 96]. Clinical trials targeting B7‐H3 have mainly focused on other solid tumors (e.g., prostate cancer, lung cancer), with less research on TC. Studies have found that B7‐H3 is significantly upregulated in high‐risk TC (e.g., poorly differentiated or undifferentiated types), and its expression level is positively correlated with tumor invasiveness, metastasis risk, and poor prognosis [64]. Similar to PD‐L1, B7‐H3 may promote tumor progression by inhibiting T‐cell function and facilitating immune evasion. Overexpression of B7‐H3 is also associated with the infiltration of immunosuppressive cells (e.g., TAMs) in the TME, further weakening anti‐tumor immune responses [94, 97]. Hińcza‐Nowak et al. [98] found that B7‐H3 expression in MTC cells was three times higher than in normal tissues. Song et al. [94] demonstrated that B7‐H3 expression was relatively weak in MTC and FTC, while PTC and ATC exhibited moderate to strong expression. Zhao's study [99] on 343 PTC patients revealed that 84.8% (291/343) had B7‐H3 positivity, with higher expression levels of B7‐H3 mRNA and protein observed in tumor tissues compared to adjacent non‐tumor tissues, suggesting its potential as an independent predictor of recurrence‐free survival.

### 3.3 CTLA‐4

CTLA‐4 is another key immune checkpoint that fine‐tunes T‐cell activation and immune tolerance. It has been used by tumors to induce an immune‐suppressive state and promote tumor growth [100]. CTLA‐4 primarily acts during the early phases of immune tolerance induction, whereas PD‐1 plays a role in maintaining long‐term tolerance [101]. Anand's study [62] showed CTLA‐4 positivity in 6.6% of PTC cases (6/90), with co‐expression of PD‐L1 and CTLA‐4 in three PTC cases, highlighting the potential of combination immunotherapy. Another study reported CTLA‐4 positivity in 12.5% (25/200) of MTC patients [102]. Moreover, CTLA‐4 expression was significantly higher in BRAF‐mutant cases compared to BRAF‐wild‐type cases [13]. Further research is needed to fully understand the mechanisms of CTLA‐4 in TC.

### 3.4 Other Immune Checkpoints

Beyond the commonly studied PD‐1/PD‐L1, B7‐H3, and CTLA‐4, other immune checkpoint molecules, including intercellular adhesion molecule 1 [103], T‐cell immunoglobulin and TIM‐3 [102], indoleamine 2,3‐dioxygenase 1 [104, 105], CD73, and CD200 [13], have also garnered attention in TC. A study with a human ATC cell line reported that the CD56hiCD16hi/lo NK cells exhibited higher PD‐1 and TIM‐3 expression while demonstrating lower cytotoxicity. Further analysis using CAL‐62 cells, which co‐expressed PD‐L1 and Gal‐9 (the ligand for TIM‐3), revealed that blocking PD‐1 and TIM‐3 significantly enhanced NK cell cytotoxicity [106]. Multi‐target immune checkpoint blockade based on personalized immune profiling could represent a promising future direction for TC immunotherapy [102]. Exploring multiple immune checkpoints is crucial for developing more effective monotherapy or combination immunotherapies, overcoming tumor resistance, and improving TC treatment by better understanding and manipulating immune responses.

## 4 Immunotherapy in Clinical Trials for TC

Immunotherapy, by restarting and sustaining the tumor immune cycle, has become one of the most promising treatment strategies in cancer therapy [107]. It has shown remarkable efficacy in reducing disease recurrence and prolonging patient survival in TC. Through various combination therapies, immunotherapy has also managed to synergistically suppress tumors while controlling treatment‐related side effects. However, challenges remain, particularly regarding resistance and adverse effects [108]. Looking ahead, the future direction of immunotherapy in TC is likely to involve combined approaches, integrating it with chemotherapy or targeted therapies. However, immune‐related adverse events (irAEs), such as hypothyroidism, Hashimoto's thyroiditis (HT), and even pituitary inflammation or adrenal insufficiency, remain unavoidable [109, 110].

### 4.1 Immune Checkpoint Inhibitors in TC

**FIGURE 2:**
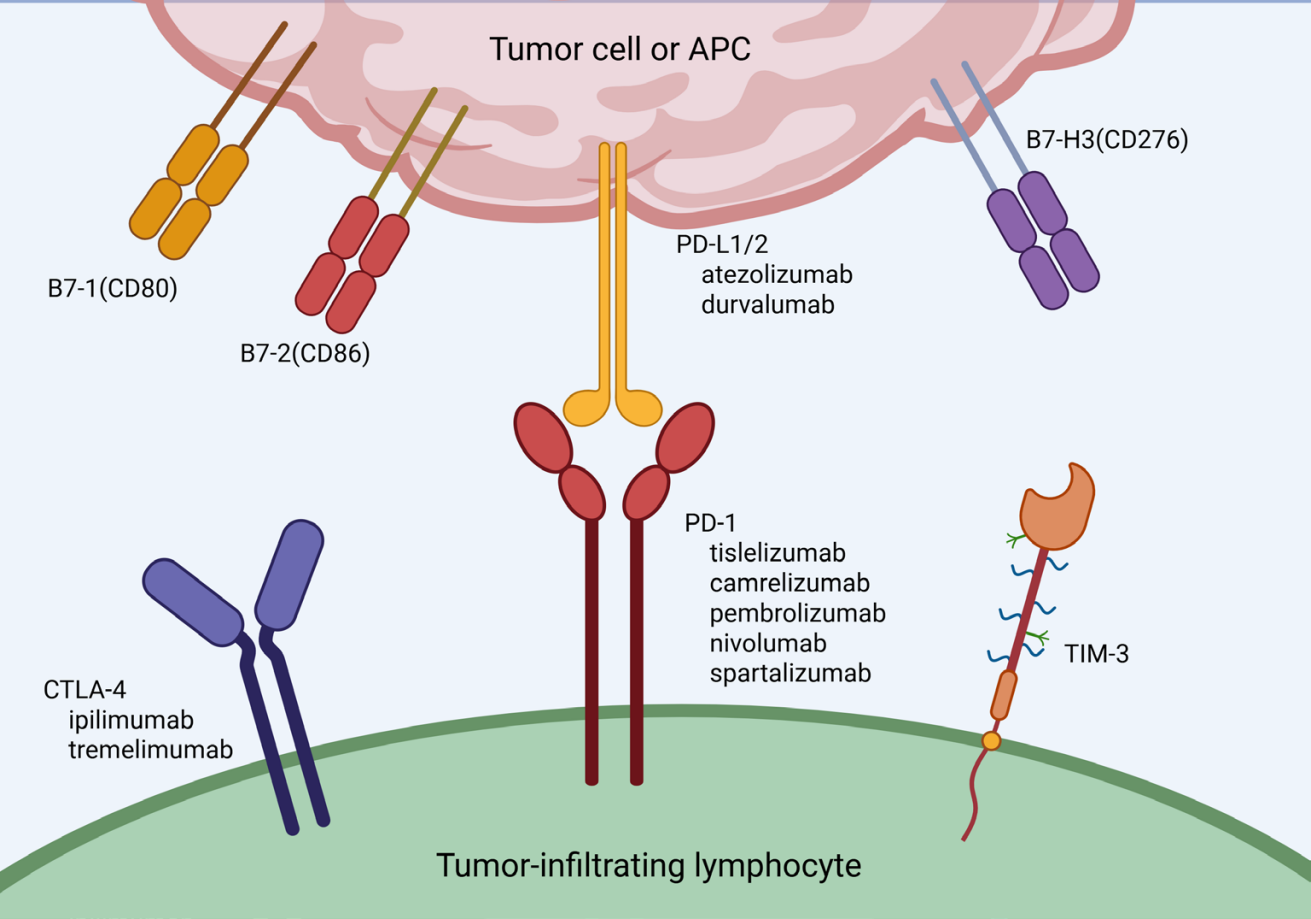
Key tumor immune checkpoint molecules and corresponding inhibitors in thyroid cancer immunotherapy. PD‐L1/2, expressed on tumor cells or APCs, binds to PD‐1 on TILs, transmitting inhibitory signals and inducing immune exhaustion. CTLA‐4 interacts with B7‐1/CD80 and B7‐2/CD86 on APCs, suppressing T‐cell activation. TIM‐3, another immune checkpoint on TILs, contributes to tumor immune evasion. B7‐H3 (CD276), minimally expressed in normal tissues but upregulated in cancer cells, inhibits T‐cell function. Common therapeutic antibodies targeting CTLA‐4, PD‐1, and PD‐L1 are listed in the figure. APC, antigen‐presenting cell; PD‐L1/2, programmed death ligand‐1/2; PD‐1, programmed death‐1; CTLA‐4, Cytotoxic T‐Lymphocyte‐Associated Protein 4; TIM‐3, T‐cell immunoglobulin and mucin domain‐3. (Figure was created with BioRender.com).

The clinical exploration of immunotherapy in TC began in 1997, with ICIs emerging as a key therapeutic approach [111]. These monoclonal antibodies target immune checkpoint proteins to block their interactions with partner proteins, effectively enhancing the immune system's ability to combat cancer. The FDA has approved several ICIs targeting different checkpoint proteins in various tumor types, including CTLA‐4 inhibitors, PD‐1 inhibitors, and PD‐L1 inhibitors [112, 113] (Figure 2). ICI monotherapy demonstrated moderate anti‐tumor activity and relatively controllable safety [77, 114, 115], while dual ICI therapy was more effective than monotherapy, with objective response rate (ORR) reaching 33% in some subtypes, but treatment‐related adverse events (AEs) increased significantly and required close monitoring [116] (Table 2).

**TABLE 2:** Results of published clinical trials on immunotherapy for TC.

Author	Treatment strategy	Trial	Phase	N	Condition/disease	Intervention	Median overall survival (OS)	Objective response rate (ORR)^a^	Treatment‐related adverse events	Antigen targeted
Janice M Mehnert et al. [114]	ICI monotherapy	NCT02054806	Phase Ib	22	PTC/FTC (failure of or intolerance to prior therapy, with PD‐L1–positive)	Pembrolizumab (10 mg/kg)/2 weeks, iv.	Not reached (95% CI, 22 months–not reached)	9% (95% CI, 1%–29%) (PR, *n* = 2)	82% (All grades); 4.5% (grade ≥ 3)	PD‐1
Jaume Capdevila et al. [115]	ICI monotherapy	NCT02404441	Phase II	42	ATC	Spartalizumab (400 mg)/4 weeks, iv.	5.9 months (95% CI, 2.4 months–not reached)	RECIST v1.1: 19% (95% CI, 8.6%–34.1%) (CR, *n* = 3; PR, *n* = 5) irRC: 24% (95% CI, 12.1%–39.5%) (CR, *n* = 3; PR, *n* = 7)	45% (All grades); 10% (grade 3/4)	PD‐1
Do‐Youn Oh et al. [77]	ICI monotherapy	NCT02628067	Phase II	103	PTC/FTC (failure of or intolerance to prior therapy)	Pembrolizumab (200 mg)/3 weeks, iv.	34.5 months (95% CI, 21.2–not reached)	6.8% (95% CI, 2.8%–13.5%) (CR, *n* = 2; PR, *n* = 5) PD‐L1 CPS ≥ 1 (*n* = 46): 8.7% (95% CI, 2.4%–20.8%) (CR, *n* = 2; PR, *n* = 2) PD‐L1 CPS < 1 (*n* = 53): 5.7% (95% CI, 1.2%–15.7%) (PR, *n* = 3)	69.9% (All grades); 14.6% (grade 3–5)	PD‐1
Sehgal Kartik et al. [116]	Dual ICI therapy	NCT03246958	Phase II	32	RAIR DTC	Nivolumab (3 mg/kg)/2 weeks, iv. Plus ipilimumab (1 mg/kg)/6 weeks, iv.	24.6 months (95% CI, 24.6–not available)	9.4% (95% CI, 2.8%–28.5%) (PR, *n* = 3) Oncocytic subtype (*n* = 6): 33.0% PDTC subtype (*n* = 5): 20.0%	81.6% (All grades); 24.5% (grade 3); 8.2% (grade 4)	PD‐1 and CTLA‐4
				10	ATC		Not reached	30% (95% CI, 6.7%–65.2%) (PR, *n* = 3)		
				7	MTC		Not reached	0%		
Dongmei Ji et al. [117]	ICI combined with targeted therapy	NCT04521348	Phase II	12	RAIR DTC	Camrelizumab (200 mg) on Day 1 of each 21‐day cycle, iv. Plus famitinib (20 mg) po.	Not mature	33.30%	The most common treatment related AEs were diarrhea (37.0%), palmoplantiplanar swelling syndrome (34.2%), hypertension (31.5%) and fatigue (31.5%).	PD‐1
				26	DTC (ineligible for ^131^I)		Not mature	44.00%		
				5	MTC		Not mature	40.00%		
				31	ATC		13.6 months	62.50%		
Jena D. French et al. [118]	ICI combined with targeted therapy	NCT02973997	Phase II	29	RAIR DTC (cohort 1: naïve to multikinase inhibitors)	Pembrolizumab (200 mg)/3 weeks, iv. Plus Lenvatinib (20 mg)/day, po.	Not reached	65.5% (PR, *n* = 19)	86.7% (grade 3); 16.7% (grade 4)	PD‐1
				25	RAIR DTC (cohort 2: had progressed on lenvatinib)	Pembrolizumab (200 mg)/3 weeks, iv. Plus Lenvatinib (dose at progression), po.	23.8 months (95% CI, 18.9–32.3)	16% (PR, *n* = 4)	56% (grade 3); 4% (grade 4)	PD‐1
Maria E. Cabanillas et al. [119]	ICI combined with targeted therapy	NCT03181100	Phase II	42	ATC	Atezolizumab combined with targeted therapy	18.23 months (95% CI, 7.79–43.24)	31% (CR, *n* = 1; PR, *n* = 12)	Not summarized	PD‐L1
				18	Cohort 1 (with a BRAF V600E mutation)	Atezolizumab (840 mg) on Day 1 and Day 15 in a 28‐day cycle, iv. Plus vemurafenib/cobimetinib, po.	43.24 months (95% CI, 16–not estimable)	50% (CR, *n* = 1; PR, *n* = 8)	Not summarized	
				21	Cohort 2 (with RAS (NRAS, KRAS, or HRAS) or NF1/2 driver mutations)	Atezolizumab (840 mg) on Day 1 and Day 15 in a 28‐day cycle, iv. Plus cobimetinib, po.	8.74 months (95% CI, 5.13–36.96)	14% (PR, *n* = 3)	Not summarized	
				3	Cohort 3 (without any of these mutations)	Atezolizumab (1200 mg)/21 days, iv. Plus bevacizumab, iv.	6.21 months (4.11–not estimable)	33% (PR, *n* = 1)	Not summarized	

Abbreviations: ATC, anaplastic thyroid carcinoma; CR, complete response; CTLA‐4, cytotoxic T‐lymphocyte–associated protein 4; DTC, differentiated thyroid carcinoma; FTC, follicular thyroid carcinoma; ICI, immune checkpoint inhibition; irRC, immune‐related Response Criteria; MTC, medullary thyroid carcinoma; PR, partial response; PTC, papillary thyroid carcinoma; RAIR, radioiodine refractory; TC, thyroid carcinoma.

^a^ Unmarked rates refer to RECIST (Response Evaluation Criteria in Solid Tumors), version 1.1.

In a phase Ib trial with PD‐L1‐positive, advanced PTC/FTC (NCT02054806) [114], pembrolizumab monotherapy demonstrated an ORR of 9%, with median overall survival (OS) not yet reached, suggesting it may offer durable survival benefits in a specific patient population, with overall manageable safety. Another multi‐cohort phase II study of pembrolizumab monotherapy (NCT02628067) [77], the ORR was 8.7% in the PD‐L1‐positive group and 5.7% in the PD‐L1‐negative group, with an overall ORR of 6.8%. Researchers suggested that future evaluations of ICI in patients with DTC should focus on biomarker‐driven patient selection or combination with other drugs to achieve higher response rates than those observed in this study [77]. However, in a phase II study (NCT02404441) [115], spartalizumab monotherapy demonstrated good clinical activity and safety in a patient population with aggressive, incurable TC and a short life expectancy. Targeted PD‐1/PD‐L1 therapy may offer a much‐needed treatment option for patients with PD‐L1‐positive advanced ATC [115]. In summary, monotherapy with ICIs can achieve some clinical response in patients with advanced or recurrent TC, but the ORR is relatively low. A moderate‐to‐high proportion of patients experience treatment‐related AEs, most of which are low‐grade, but there are still 3–5 grade reactions that require intervention. This strategy is more likely to be suitable for patients with PD‐L1‐positive expression or immune‐responsive subpopulations.

Sehgal Kartik et al. [116] investigate the efficacy and safety of ICIs in a Phase II clinical trial (NCT03246958). Key findings indicate that for the radioiodine refractory (RAIR) DTC cohort, the median OS was 24.6 months with an ORR of 9.4%. For the ATC cohort, the median OS was not reached, and the objective response rate was 30%. This demonstrates promising survival outcomes in both RAIR DTC and ATC, particularly with a notable response in the oncocytic subtype of RAIR DTC. However, the trial had a treatment‐related adverse event incidence rate of 81.6%. Grade 3 and grade 4 adverse events occurred in 24.5% and 8.2% of cases, respectively. Dual ICIs warrant investigation in larger ATC‐focused clinical trials and hold promise as a new treatment paradigm for these patients.

Case reports have also highlighted the potential benefits of ICIs in specific TC patient populations. For example, 13 patients with inoperable ATC treated with DTP showed a 1‐year survival rate of 38%, although nearly half (46%) experienced irAEs [120]. Additionally, patients with metastatic MTC who had developed resistance to tyrosine kinase inhibitors (TKIs) benefited from ICIs after alkylating agent treatment induced high tumor mutation burdens [121].

### 4.2 Combination Strategies With ICIs

**FIGURE 3:**
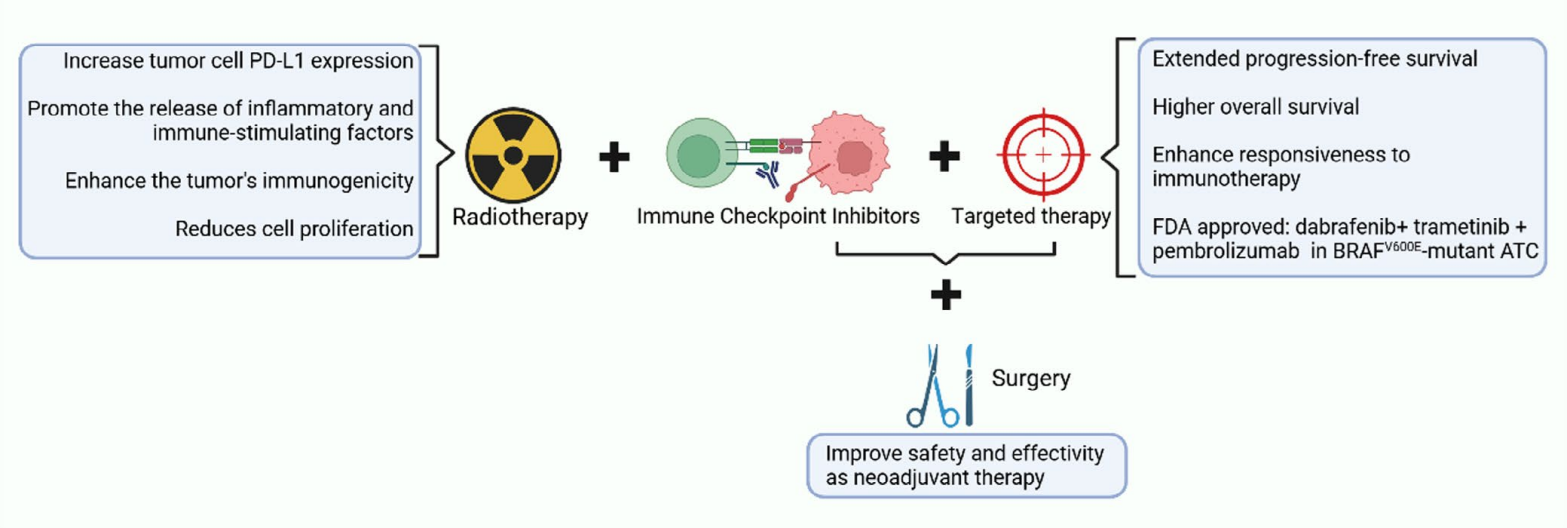
Synergistic therapeutic effects of immune checkpoint inhibitors (ICIs) with radiotherapy, targeted therapy, and surgery. The combination of ICIs with radiotherapy, targeted therapy, or surgery enhances therapeutic efficacy through multiple mechanisms. Radiotherapy upregulates tumor cell PD‐L1 expression, promotes the release of inflammatory cytokines and immunostimulatory factors, and reduces tumor cell proliferation, thereby potentiating ICI efficacy. The integration of ICIs with targeted therapy significantly improves progression‐free survival, overall survival, and responsiveness to immunotherapy. Additionally, neoadjuvant therapy combining ICIs and targeted therapy has improved the safety and efficacy of surgical intervention. (Figure was created with BioRender.com).

Although ICIs have revolutionized cancer treatment, not all patients respond effectively to monotherapy. To overcome this challenge, researchers are actively exploring combination therapies to enhance efficacy and improve resistance, particularly in advanced TC or cases where other single‐agent treatments have failed [122]. These combinations are designed to enhance the ability of the immune system to combat cancer more effectively than monotherapies [123]. Combining ICI with other therapies, such as targeted therapies, radiotherapy, or surgery, has shown promising results (Figure 3).

Current clinical trial evidence indicates that, compared to monotherapy with ICIs, the combination of ICIs with targeted therapy provides significant survival benefits for advanced TC, particularly for RAIR‐DTC and ATC (Table 2). In two phase II clinical trials, camrelizumab (NCT04521348) [117] and atezolizumab (NCT03181100) [119] combined with targeted therapy increased the ORR in the ATC cohort to 62.5% and 31%, respectively, with a median OS of 43.24 months and an ORR of 50% in the BRAF V600E mutation ATC cohort [119]. The combination of camrelizumab and the multi‐kinase inhibitor famitinib (a kinase inhibitor) for RAIR‐DTC achieved an ORR of 33.3% [119]. While Jena D. French et al. (NCT02973997) [118] further revealed that lenvatinib combined with pembrolizumab can enhance durability in RAIR‐DTC patients who have not previously received lenvatinib treatment. Additionally, for patients who have progressed on lenvatinib treatment, adding pembrolizumab may be a viable salvage therapy. For DTC (ineligible for ^131^I) and MTC patients, the combination of camrelizumab and famitinib achieved an ORR of 44.00% and 40.00%, respectively. Future efforts should focus on patient stratification guided by biomarkers and mechanism‐driven combination regimens to further expand the population benefiting from ICIs combined with targeted therapy and improve the treatment window. Multiple retrospective studies have also confirmed the efficacy of combination therapy in achieving higher OS and treating patients who are resistant to traditional therapies [124, 125, 126, 127].

Combination therapy can also be used as neoadjuvant treatment. A single‐center retrospective study [128] assessing 18 patients with unresectable ATC found that combining kinase inhibitors and anti‐PD‐1 antibody as neoadjuvant therapy was both safe and effective. Approximately 38.9% (7/18) of patients underwent surgical resection after treatment, with a median overall survival of 14.0 months and a 12‐month survival rate of 55.6%.

For patients with specific genetic mutations, targeted therapy combined with ICIs has shown promising results. Research has found that PD‐L1 expression in PTC and ATC is significantly associated with BRAF^V600E^ mutations, suggesting that combined BRAF^V600E^ and PD‐1/PD‐L1 axis targeting may have clinical potential [66]. Studies in mice and single‐cell RNA‐sequencing analysis of tumor samples from treated patients indicated that famitinib combined with anti‐PD‐1 antibodies could enhance the development of early tertiary lymphoid structures in ATC, making it more responsive to immunotherapy [16]. The combination of targeted therapy and ICIs appears to be effective as neoadjuvant therapy for BRAF^V600E^‐mutated ATC [129]. The FAST Multidisciplinary Group Consensus Statement recommends combining dabrafenib (selective BRAF kinase inhibitors), trametinib (MEK1/2 inhibitors), and pembrolizumab for BRAF^V600E^‐variant ATC patients, which may offer significant survival benefits over using dabrafenib and trametinib alone [130].

Several studies have reported that radiotherapy can increase tumor cell PD‐L1 expression, promote the release of inflammatory and immune‐stimulating factors, and enhance the tumor's immunogenicity, thus improving the effects of immunotherapy [131, 132]. Therefore, numerous studies have investigated the combination of ICIs with radiotherapy. In vitro studies have shown that adding atezolizumab to radiation‐treated ATC cells significantly reduces cell proliferation [133]. Xing [134] reported a case in which radiation combined with tislelizumab successfully treated an ATC patient with good tolerance. Goh [135] described the combination of radiotherapy and pembrolizumab in a PD‐L1 high‐expressing ATC patient with a combined positive score of over 70%, resulting in a complete response of both local and distant disease. However, a phase I clinical trial using durvalumab combined with tremelimumab and image‐guided stereotactic body radiation therapy for metastatic ATC failed to improve overall survival, with only one out of 12 patients surviving after 1 year [136]. Further randomized controlled trials are needed to fully understand whether combining radiotherapy with immunotherapy offers potential benefits and to optimize treatment strategies for different types of TC.

### 4.3 Adoptive Immunotherapy

**FIGURE 4:**
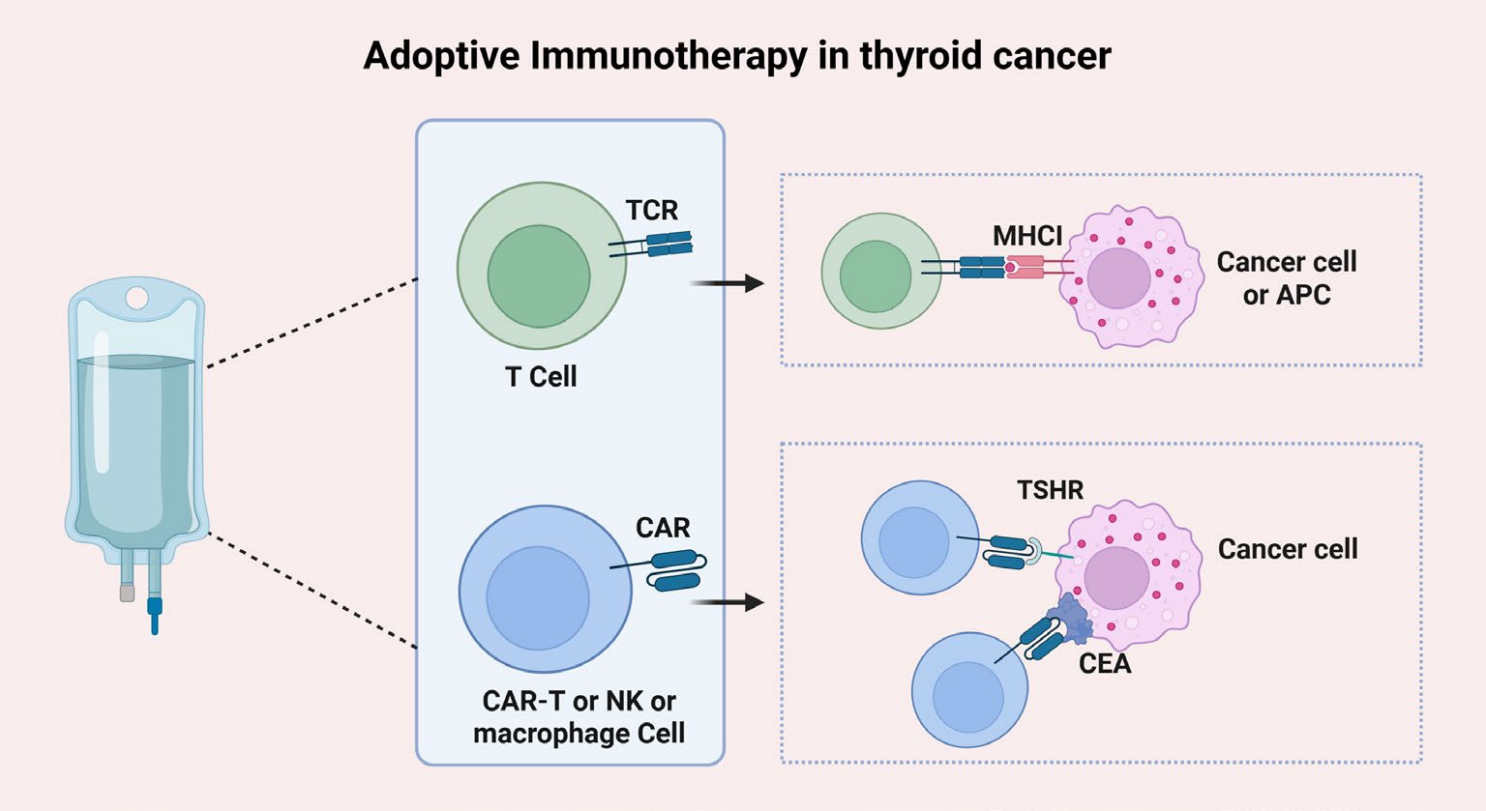
Current adoptive immunotherapy in TC. TCR and CAR therapies are emerging strategies in thyroid cancer immunotherapy. TSHR and CEA have been identified as promising CAR targets in TC. APC, antigen‐presenting cell; CAR: chimeric antigen receptor; CEA: carcinoembryonic antigen; NK: natural killer cells; T cell: T lymphocytes; TCR: T‐cell receptor; TSHR: thyrotropin receptor (Figure was created with BioRender.com).

Adoptive immunotherapy involves extracting immune cells from the patient, expanding or genetically modifying them to enhance anti‐tumor activity, and then re‐infusing them into the patient to bolster the immune system. Several approaches to adoptive immunotherapy are currently being explored in TC (Figure 4).

#### 4.3.1 Chimeric Antigen Receptor T‐Cell Therapy

CAR‐T therapy is a groundbreaking treatment that enables T cells to recognize tumor antigens in an HLA‐independent manner. While it has shown significant success in hematologic malignancies, its application in solid tumors like TC remains limited due to TME complexity and antigen heterogeneity [137, 138]. TSHR, expressed on the basal membrane of thyroid follicular cells, is a key target in CAR‐T research. Li's team [139] assessed the safety and efficacy of anti‐TSHR CAR‐T in vitro and in vivo. In another case report, TSHR + CD19 CAR‐T successfully treated refractory and relapsed TC patients, achieving partial remission [140]. Additionally, CEA is another potential target. CAR‐T targeting CEA has shown promise for treating metastatic MTC [141], though concerns about off‐target effects due to CEA expression in normal tissues remain.

#### 4.3.2 T‐Cell Receptor (TCR) Therapy

TCR therapy involves modifying T cells to express receptors that recognize specific tumor antigens presented on the cancer cell surface by the major histocompatibility complex [142]. This approach expands the range of targeted tumor antigens but faces challenges related to complex production processes, non‐specific cytotoxicity, and improving T‐cell persistence [142, 143]. Cui et al. [144] using TCR high‐throughput sequencing with small sample size (*n* = 6) confirmed that intratumor heterogeneity of the T‐cell quantity and TCR repertoire truly existed in PTC, and the number of CD3+ TILs was negatively associated with TCR clonality in PTC.

#### 4.3.3 NK Cell Therapy

NK cells can be isolated, expanded, and sometimes genetically modified to enhance their tumor‐targeting capacity. TSHR‐CAR‐modified NK‐92 cells exhibited enhanced cytotoxicity against TSHR‐positive DTC cell lines along with increased degranulation and cytokine release, providing a promising option for advancing immunotherapy in DTC [145].

#### 4.3.4 Tumor‐Infiltrating Lymphocyte Therapy

TILs possess diverse TCR clonality, excellent tumor‐targeting ability, and low off‐target toxicity [146]. A study on PTC used artificial intelligence models to analyze TIL density in tumor tissues, categorizing tumors into three immune phenotypes: immune desert (48%), immune exclusion (34%), and inflammation (18%) [147]. TIL therapy has already demonstrated potential in treating other solid tumors, such as melanoma [148]. A phase II trial (NCT03449108), expected to be completed by June 2025, is currently investigating the efficacy of autologous TILs (LT‐145 or LN‐145‐S1) in patients with ATC and other cancers.

## 5 New Explorations in Immunotherapy for TC

Recent advancements in immunotherapy for TC have significantly expanded our understanding beyond the identification of specific tumor markers. Researchers have leveraged advanced experimental technologies and online databases to uncover promising biomarkers that not only shed light on the molecular mechanism driving TC progression but also suggest novel strategies for optimizing immunotherapy. These studies enhance our understanding of tumor immunity in TC, improve the efficacy of immune therapies, and provide potential biomarkers for predicting treatment responses, ultimately aiding in the development of personalized treatment strategies [149].

### 5.1 N6‐Methyladenosine

N6‐methyladenosine (m6A) is a prevalent RNA modification in eukaryotes, playing a dual role in cancer by influencing the immune microenvironment, genomic stability, and non‐coding RNA networks [150]. In TC, m6A modifications regulate RNA metabolism, impacting tumorigenesis, invasion, and immune evasion. Key regulatory factors (e.g., METTL3, YTHDF2) and downstream target genes (e.g., ACSM5) are of significant clinical relevance [151, 152, 153]. METTL3, by mediating the m6A modification of target genes like ACSM5, suppresses the proliferation, migration, and invasion of TC cells [152]. Moreover, the loss of METTL3 can promote TC dedifferentiation, leading to worse patient prognosis [154].

M6A modification may influence immune cell infiltration within the TC microenvironment by regulating the expression of immune‐related genes. A study revealed that patients with low m6A scores exhibited higher expression of immune checkpoints such as LAG3 and CTLA‐4, suggesting poorer responses to immunotherapy [155]. Similarly, Zhou et al. [156] found that TC patients with low m6A scores had higher PD‐L1 and CTLA‐4 expression. These findings suggest that m6A scores could serve as potential indicators of immunotherapy efficacy, with m6A modification patterns offering valuable insights for guiding personalized immunotherapy. Cai [157] identified four m6A patterns, with the m6A cluster‐mc3 subtype exhibiting low m6A scores, high copy number burden, and poor prognosis. Xia et al. [158] constructed a risk model based on 16 m6A‐related genes, revealing that high‐risk groups had lower immune infiltration, suggesting that m6A influences tumor progression via immune pathways.

The role of m6A‐related long non‐coding RNAs (lncRNAs) is also noteworthy. Su's team [159] classified TC patients into three clusters based on m6A‐related lncRNAs, with Cluster 2 showing higher expression of PD‐L1 and CTLA‐4 and better survival. A risk model based on 11 key lncRNAs accurately assessed immune status and risk, with experimental validation showing that reducing representative lncRNAs inhibited TC cell proliferation and migration. Chen et al. [160] identified two m6A‐related lncRNAs, LINC02471 and DOCK9‐DT, linked to immune cell infiltration, offering potential targets for immunotherapy.

### 5.2 Sialic Acid‐Binding Immunoglobulin‐Type Lectin (SIGLECs)

SIGLECs are receptors on leukocyte membranes that recognize sialylated glycoproteins, helping immune cells distinguish between “self” and “non‐self.” This function influences immune responses and the TME. In the TME, SIGLECs promote immune evasion through mechanisms similar to the PD‐1/PD‐L1 pathway, which has garnered significant attention. However, the exact role of SIGLECs in TC remains unclear [161, 162]. Among the SIGLEC family, SIGLEC10 and SIGLEC15 have emerged as promising immunotherapy targets.

SIGLEC15 expression may be epigenetically regulated, affecting immune cell infiltration and shaping the immune characteristics of TC. Hou's study [162] indicated that m6A methylation regulators influence SIGLEC15 expression, providing a potential mechanism for its aberrant expression in cancer. Additionally, high SIGLEC15 expression in ATC cells may foster an immunosuppressive TME by enhancing proteasome activity, while anti‐SIGLEC15 antibodies can inhibit tumor growth by boosting T‐cell‐mediated cytotoxicity [163]. In an ATC mouse model, these antibodies increased infiltration of M1 macrophages, NK cells, and CD8+ T cells, while reducing myeloid‐derived suppressor cells. The treatment also promoted the secretion of IFN‐γ and IL‐2, reversing the immunosuppressive TME and enhancing anti‐tumor immune responses [163].

The expression of SIGLEC15 is associated with malignant progression in TC. High SIGLEC15 expression correlates with increased TME cell interactions and is linked to extrathyroidal extension, lymph node metastasis, and immune exhaustion [162, 163]. Co‐expression of SIGLEC10 and SIGLEC15 in PTC tumor cells and stroma is a significant predictor of recurrence risk [164]. Therefore, blocking SIGLEC15 may benefit TC patients [162]. Additionally, SIGLEC15 expression is inversely correlated with key DNA damage repair deficiencies, suggesting that different SIGLEC15 expression subgroups may exhibit distinct sensitivities to drugs [162]. Collectively, the expression patterns of SIGLEC family members, particularly SIGLEC10 and SIGLEC15, and their associations with the immune microenvironment and prognosis, open new avenues for next‐generation immunotherapy in TC.

### 5.3 Thyroiditis and TC

The co‐occurrence of HT and PTC raises ongoing debate about whether HT provides a protective or promoting effect on PTC. The underlying biological mechanisms remain unclear [165, 166]. Several studies have explored the impact of thyroiditis on PTC immunotherapy outcomes. In a cohort study of 9210 PTC patients, 19% were diagnosed with HT [167]. Some studies suggest that HT may increase the risk of TC [168, 169], while others indicate that TC patients with HT have better prognostic outcomes compared to those without HT [167, 170].

Li et al. [170] reported that HT exhibited significantly higher immune scores and CD8+ T cell abundance compared to those without HT, with the high abundance of CD8+ T cells being positively correlated with disease‐free survival in PTC patients. Moreover, PD‐1 gene expression was notably higher in the HT group than in the non‐HT group [170]. Ma's team [171] used data from 140,456 cells across 11 patients to investigate the role of HT in shaping the PTC tumor immune microenvironment. They found that HT‐associated cell populations, enriched in thyroid hormone pathways such as mTE3, nTE0, and nTE2, created a TSH‐suppressive environment, positively influencing PTC progression. Furthermore, Pani's team [172] proposed that iodine‐induced thyroiditis (IET) could serve as an adjunct therapy to modulate the immune system and improve responses to ICIs. Their research showed that in mouse models with concurrent IET and PTC, ICI treatment reduced PTC incidence, while no effect was observed in models with pre‐existing IET or without IET.

The chronic inflammation associated with HT leads to lymphocyte infiltration, which influences TC development. Preliminary studies, using single‐cell RNA sequencing analysis of peritumoral and intra‐tumoral immune cells in TC that are coexistent with thyroiditis, could be of paramount importance to elucidate their functions [173].

## 6 Conclusions and Future Perspectives

Recent advances in immunotherapy have demonstrated significant potential in treating advanced TC, particularly for aggressive subtypes such as ATC and refractory DTC. PD‐1/PD‐L1 inhibitors, among the most extensively studied ICIs, not only modulate the TME and induce T‐cell exhaustion but also influence tumor development through metabolic reprogramming and protein modification. CTLA‐4, which acts at early stages of immune tolerance, attenuates T‐cell responses and induce T‐cell exhaustion. B7‐H3, frequently overexpressed in aggressive subtypes, has been widely reported to promote oncogenesis, angiogenesis, invasion, and metastasis through diverse mechanisms. Beyond conventional immune checkpoints, several novel biomolecules offer promising therapeutic avenues for TC. These include SIGLEC15, which remodels the immunosuppressive tumor microenvironment and is targetable with blocking antibodies; DPP4 (CD26), whose inhibition reverses CD8^+^ T‐cell exhaustion and disrupts the EMT‐promoting IL13‐IL13RA2 axis; and SPP1^+^ macrophage‐driven axes (SPP1‐CD44/MIF‐CD74) that mediate T cell suppression. Additionally, m6A RNA modification regulators (e.g., METTL3, YTHDF2) influence immune checkpoint expression and predict immunotherapy response, while adoptive therapy targets like TSHR and CEA enable CAR‐T/NK cell strategies for refractory disease. Together, these targets may address key resistance mechanisms in ATC and warrant biomarker‐driven clinical validation, particularly for anaplastic and poorly differentiated subtypes. Furthermore, combination strategies that integrate ICIs with targeted therapies (e.g., BRAF/MEK inhibitors) or adoptive cell therapies (e.g., CAR‐T) are under investigation to overcome resistance and enhance efficacy [174].

Despite progress, challenges persist, including tumor heterogeneity, immune evasion mechanisms, and limited durable responses observed in clinical trials. Future research may focus on identifying predictive biomarkers (e.g., PD‐L1 expression, immune risk scores) to better stratify patients, optimizing combination regimens to mitigate irAEs, and exploring novel immunotherapeutic approaches, such as targeting TAMs heterogeneity (e.g., tissue‐resident TRM‐TAMs vs. monocytic‐derived MDM‐TAMs) [175]. Additionally, leveraging preclinical models and multi‐omics data will be crucial for unraveling the complex interactions within the immune microenvironment and understanding the epigenetic regulation of TC. As clinical trials progress and our understanding of tumor‐immune interactions deepens, immunotherapy is poised to redefine the therapeutic landscape for advanced TC.

## Author Contributions

Q.Z. and F.W. conducted the literature research and drafted the manuscript. W.M. and N.L. drew the pictures according to the literature data. Y.L. and P.Z. revised the paper and made critical interpretations of the literature data. All authors have read and agreed to the published version of the manuscript.

## Funding

Yihan Lu was supported by the National Nature Science Foundation of China (grant number 82200878) and Dalian Science and Technology Bureau (grant number 2023RQ013).

## Conflicts of Interest

The authors declare no conflicts of interest.

## Data Availability

The authors have nothing to report.

## References

[1] R. L. Siegel , K. D. Miller , H. E. Fuchs , and A. Jemal , “Cancer Statistics, 2021,” CA: A Cancer Journal for Clinicians 71, no. 1 (2021): 7–33. 33433946 10.3322/caac.21654

[2] GBD 2019 Diseases and Injuries Collaborators , “Global Burden of 369 Diseases and Injuries in 204 Countries and Territories, 1990‐2019: A Systematic Analysis for the Global Burden of Disease Study 2019,” Lancet 396, no. 10258 (2020): 1204–1222. 33069326 10.1016/S0140-6736(20)30925-9 PMC7567026

[3] Y. Yang , X. Bai , J. Lu , R. Zou , R. Ding , and X. Hua , “Assessment of Five Typical Environmental Endocrine Disruptors and Thyroid Cancer Risk: A Meta‐Analysis,” Frontiers in Endocrinology (Lausanne) 14 (2023): 1283087. 10.3389/fendo.2023.1283087 PMC10643203 38027118

[4] Z. W. Baloch , S. L. Asa , J. A. Barletta , et al., “Overview of the 2022 WHO Classification of Thyroid Neoplasms,” Endocrine Pathology 33, no. 1 (2022): 27–63. 35288841 10.1007/s12022-022-09707-3

[5] L. G. Yang , Z. G. Yang , J. Zhang , and Y. L. Fu , “Effects of (131)I and TSH Suppression Therapy on METTL3, METTL14 Levels and Recurrence in Thyroid Cancer,” American Journal of Cancer Research 15, no. 1 (2025): 42–58. 39949928 10.62347/THJB4749 PMC11815380

[6] S. Filetti , C. Durante , D. M. Hartl , et al., “ESMO Clinical Practice Guideline Update on the Use of Systemic Therapy in Advanced Thyroid Cancer,” Annals of Oncology 33, no. 7 (2022): 674–684. 35491008 10.1016/j.annonc.2022.04.009

[7] Q. Wang , G. Pan , Y. Zhang , Y. Ni , Y. Mu , and D. Luo , “Emerging Insights Into Thyroid Cancer From Immunotherapy Perspective: A Bibliometric Analysis,” Human Vaccines & Immunotherapeutics 20, no. 1 (2024): 2403170. 39294892 10.1080/21645515.2024.2403170 PMC11657091

[8] G. P. Dunn , A. T. Bruce , H. Ikeda , L. J. Old , and R. D. Schreiber , “Cancer Immunoediting: From Immunosurveillance to Tumor Escape,” Nature Immunology 3, no. 11 (2002): 991–998. 12407406 10.1038/ni1102-991

[9] C. C. Zebley , D. Zehn , S. Gottschalk , and H. Chi , “T Cell Dysfunction and Therapeutic Intervention in Cancer,” Nature Immunology 25, no. 8 (2024): 1344–1354. 39025962 10.1038/s41590-024-01896-9 PMC11616736

[10] N. M. Anderson and M. C. Simon , “The Tumor Microenvironment,” Current Biology 30, no. 16 (2020): R921–r925. 32810447 10.1016/j.cub.2020.06.081 PMC8194051

[11] X. Li , J. Jian , A. Zhang , J. M. Xiang , J. Huang , and Y. Chen , “The Role of Immune Cells and Immune Related Genes in the Tumor Microenvironment of Papillary Thyroid Cancer and Their Significance for Immunotherapy,” Scientific Reports 14, no. 1 (2024): 18125. 39103463 10.1038/s41598-024-69187-9 PMC11300445

[12] M. Li , J. Zhang , Z. Zhang , et al., “Identification of Transcriptional Pattern Related to Immune Cell Infiltration With Gene co‐Expression Network in Papillary Thyroid Cancer,” Frontiers in Endocrinology (Lausanne) 13 (2022): 721569. 10.3389/fendo.2022.721569 PMC8854657 35185791

[13] A. Mohanty , M. Afkhami , A. Reyes , et al., “Exploring Markers of Immunoresponsiveness in Papillary Thyroid Carcinoma and Future Treatment Strategies,” Journal for Immunotherapy of Cancer 12, no. 7 (2024): e008505. 39074963 10.1136/jitc-2023-008505 PMC11288153

[14] Z. Wang , X. Ji , Y. Zhang , et al., “Interactions Between LAMP3+ Dendritic Cells and T‐Cell Subpopulations Promote Immune Evasion in Papillary Thyroid Carcinoma,” Journal for Immunotherapy of Cancer 12, no. 5 (2024): e008983. 38816233 10.1136/jitc-2024-008983 PMC11141193

[15] S. Li , Z. Chen , M. Liu , et al., “Immunophenotyping With High‐Dimensional Flow Cytometry Identifies Treg Cell Subsets Associated With Recurrence in Papillary Thyroid Carcinoma,” Endocrine‐Related Cancer 31, no. 3 (2024): e230240. 38214937 10.1530/ERC-23-0240

[16] P. Z. Han , W. D. Ye , P. C. Yu , et al., “A Distinct Tumor Microenvironment Makes Anaplastic Thyroid Cancer More Lethal but Immunotherapy Sensitive Than Papillary Thyroid Cancer,” JCI Insight 9, no. 8 (2024): e173712. 38478516 10.1172/jci.insight.173712 PMC11141884

[17] Q. Hao , R. Li , H. Li , et al., “Dynamics of the Γδtcr Repertoires During the Dedifferentiation Process and Pilot Implications for Immunotherapy of Thyroid Cancer,” Advanced Science (Weinheim) 11, no. 13 (2024): e2306364. 10.1002/advs.202306364 PMC10987121 38286670

[18] X. Gao , Y. Le , C. Geng , Z. Jiang , G. Zhao , and P. Zhang , “DPP4 Is a Potential Prognostic Marker of Thyroid Carcinoma and a Target for Immunotherapy,” International Journal of Endocrinology 2022 (2022): 5181386. 36467461 10.1155/2022/5181386 PMC9715318

[19] R. Jing , N. Wu , Q. Zhang , et al., “DPP4 Promotes an Immunoenhancing Tumor Microenvironment Through Exhausted CD8+ T Cells With Activating IL13‐IL13RA2 Axis in Papillary Thyroid Cancer,” International Immunopharmacology 145 (2025): 113760. 39662266 10.1016/j.intimp.2024.113760

[20] G. Giovanni , F. Roberta , B. Cristina , et al., “Role of Macrophage Targeting in the Antitumor Activity of Trabectedin,” Cancer Cell 23, no. 2 (2013): 249–262. 23410977 10.1016/j.ccr.2013.01.008

[21] Y. Pan , Y. Yu , X. Wang , and T. Zhang , “Tumor‐Associated Macrophages in Tumor Immunity,” Frontiers in Immunology 11 (2020): 583084. 33365025 10.3389/fimmu.2020.583084 PMC7751482

[22] W. Qing , W. Y. Fang , L. Ye , et al., “Density of Tumor‐Associated Macrophages Correlates With Lymph Node Metastasis in Papillary Thyroid Carcinoma,” Thyroid 22, no. 9 (2012): 905–910. 22870901 10.1089/thy.2011.0452 PMC3429273

[23] X. Xiang , J. Wang , D. Lu , and X. Xu , “Targeting Tumor‐Associated Macrophages to Synergize Tumor Immunotherapy,” Signal Transduction and Targeted Therapy 6, no. 1 (2021): 75. 33619259 10.1038/s41392-021-00484-9 PMC7900181

[24] N. Cheng , X. Bai , Y. Shu , O. Ahmad , and P. Shen , “Targeting Tumor‐Associated Macrophages as an Antitumor Strategy,” Biochemical Pharmacology 183 (2021): 114354. 33279498 10.1016/j.bcp.2020.114354

[25] Y. Wang , D. Wang , L. Yang , and Y. Zhang , “Metabolic Reprogramming in the Immunosuppression of Tumor‐Associated Macrophages,” Chinese Medical Journal 135, no. 20 (2022): 2405–2416. 36385099 10.1097/CM9.0000000000002426 PMC9945195

[26] M. Liu , Z. X. Zhang , J. H. Wang , et al., “Immunomodulatory and Anti‐Ovarian Cancer Effects of Novel Astragalus Polysaccharide Micelles Loaded With Podophyllotoxin,” International Journal of Biological Macromolecules 290 (2025): 138960. 39708884 10.1016/j.ijbiomac.2024.138960

[27] S. Yan and G. Wan , “Tumor‐Associated Macrophages in Immunotherapy,” FEBS Journal 288, no. 21 (2021): 6174–6186. 33492779 10.1111/febs.15726

[28] T. Kimura , M. Kruhlak , L. Zhao , E. Hwang , L. Fozzatti , and S. Y. Cheng , “Combinatory Actions of Cytokines Induce M2‐Like Macrophages in Anaplastic Thyroid Cancer,” American Journal of Cancer Research 14, no. 12 (2024): 5812–5825. 39803637 10.62347/QUWQ3794 PMC11711523

[29] A. Jaroszewski , R. C. Geysels , X. Volpini , et al., “Anaplastic Thyroid Cancer Cell‐Secreted TGFβ1 Plays a Key Role in Inducing Macrophage Polarization of Human Monocytes,” American Journal of Cancer Research 14, no. 7 (2024): 3626–3638. 39113863 10.62347/BHFA4606 PMC11301286

[30] P. Zongfu , B. Lisha , L. Xixuan , et al., “IL2RA(+)VSIG4(+) Tumor‐Associated Macrophage Is a Key Subpopulation of the Immunosuppressive Microenvironment in Anaplastic Thyroid Cancer,” Biochimica et Biophysica Acta. Molecular Basis of Disease 1869, no. 1 (2022): 166591. 36328145 10.1016/j.bbadis.2022.166591

[31] L. M. Palacios , V. Peyret , M. E. Viano , et al., “TIM3 Expression in Anaplastic‐Thyroid‐Cancer‐Infiltrating Macrophages: An Emerging Immunotherapeutic Target,” Biology‐Basel 11, no. 11 (2022): 1609. 36358310 10.3390/biology11111609 PMC9687546

[32] Y. Li , Z. Wang , F. Lu , et al., “Novel T Cell Exhaustion Gene Signature to Predict Prognosis and Immunotherapy Response in Thyroid Carcinoma From Integrated RNA‐Sequencing Analysis,” Scientific Reports 14, no. 1 (2024): 8375. 38600248 10.1038/s41598-024-58419-7 PMC11006682

[33] D. I. Gabrilovich , V. Bronte , S. H. Chen , et al., “The Terminology Issue for Myeloid‐Derived Suppressor Cells,” Cancer Research 67, no. 1 (2007): 425–426. 17210725 10.1158/0008-5472.CAN-06-3037 PMC1941787

[34] Z. Hao , R. Li , Y. Wang , S. Li , Z. Hong , and Z. Han , “Landscape of Myeloid‐Derived Suppressor Cell in Tumor Immunotherapy,” Biomarker Research 9, no. 1 (2021): 77. 34689842 10.1186/s40364-021-00333-5 PMC8543853

[35] L. A. Elliott , G. A. Doherty , K. Sheahan , and E. J. Ryan , “Human Tumor‐Infiltrating Myeloid Cells: Phenotypic and Functional Diversity,” Frontiers in Immunology 8 (2017): 86. 28220123 10.3389/fimmu.2017.00086 PMC5292650

[36] K. Rabold , M. Zoodsma , I. Grondman , et al., “Reprogramming of Myeloid Cells and Their Progenitors in Patients With Non‐Medullary Thyroid Carcinoma,” Nature Communications 13, no. 1 (2022): 6149. 10.1038/s41467-022-33907-4 PMC9579179 36257966

[37] P. Zhang , H. Guan , S. Yuan , et al., “Targeting Myeloid Derived Suppressor Cells Reverts Immune Suppression and Sensitizes BRAF‐Mutant Papillary Thyroid Cancer to MAPK Inhibitors,” Nature Communications 13, no. 1 (2022): 1588. 10.1038/s41467-022-29000-5 PMC8948260 35332119

[38] A. Kotwal , M. P. Gustafson , S. Bornschlegl , A. B. Dietz , D. Delivanis , and M. Ryder , “Circulating Immunophenotypes Are Potentially Prognostic in Follicular Cell‐Derived Thyroid Cancer,” Frontiers in Immunology 14 (2023): 1325343. 38235146 10.3389/fimmu.2023.1325343 PMC10792034

[39] M. Boruah , S. Agarwal , R. A. Mir , et al., “Unravelling the Reasons Behind Limited Response to Anti‐PD Therapy in ATC: A Comprehensive Evaluation of Tumor‐Infiltrating Immune Cells and Checkpoints,” Endocrine Pathology 35, no. 4 (2024): 419–431. 39477894 10.1007/s12022-024-09832-1

[40] B. Xu , L. Zhang , R. Setoodeh , et al., “Prolonged Survival of Anaplastic Thyroid Carcinoma Is Associated With Resectability, Low Tumor‐Infiltrating Neutrophils/Myeloid‐Derived Suppressor Cells, and Low Peripheral Neutrophil‐To‐Lymphocyte Ratio,” Endocrine 76, no. 3 (2022): 612–619. 35149932 10.1007/s12020-022-03008-9 PMC10173871

[41] V. Gunda , B. Gigliotti , D. Ndishabandi , et al., “Combinations of BRAF Inhibitor and Anti‐PD‐1/PD‐L1 Antibody Improve Survival and Tumour Immunity in an Immunocompetent Model of Orthotopic Murine Anaplastic Thyroid Cancer,” British Journal of Cancer 119, no. 10 (2018): 1223–1232. 30327563 10.1038/s41416-018-0296-2 PMC6251038

[42] F. Zhang , X. Yu , Z. Lin , et al., “Using Tumor‐Infiltrating Immune Cells and a ceRNA Network Model to Construct a Prognostic Analysis Model of Thyroid Carcinoma,” Frontiers in Oncology 11 (2021): 658165. 34141614 10.3389/fonc.2021.658165 PMC8204697

[43] Z. Yang , X. Wei , Y. Pan , et al., “A New Risk Factor Indicator for Papillary Thyroid Cancer Based on Immune Infiltration,” Cell Death & Disease 12, no. 1 (2021): 51. 33414407 10.1038/s41419-020-03294-z PMC7791058

[44] M. Amanullah , M. Pan , K. Lu , et al., “Tumor‐Infiltrating Immune Cell Landscapes in the Lymph Node Metastasis of Papillary Thyroid Cancer,” Current Oncology 30, no. 3 (2023): 2625–2641. 36975413 10.3390/curroncol30030200 PMC10046895

[45] Z. Xie , X. Li , Y. He , et al., “Immune Cell Confrontation in the Papillary Thyroid Carcinoma Microenvironment,” Frontiers in Endocrinology (Lausanne) 11 (2020): 570604. 10.3389/fendo.2020.570604 PMC7642595 33193087

[46] S. Shao , Q. Xu , X. Yu , R. Pan , and Y. Chen , “Dipeptidyl Peptidase 4 Inhibitors and Their Potential Immune Modulatory Functions,” Pharmacology & Therapeutics 209 (2020): 107503. 32061923 10.1016/j.pharmthera.2020.107503 PMC7102585

[47] X. He and C. Xu , “Immune Checkpoint Signaling and Cancer Immunotherapy,” Cell Research 30, no. 8 (2020): 660–669. 32467592 10.1038/s41422-020-0343-4 PMC7395714

[48] Y. Zhang and J. Zheng , “Functions of Immune Checkpoint Molecules Beyond Immune Evasion,” Advances in Experimental Medicine and Biology 1248 (2020): 201–226. 32185712 10.1007/978-981-15-3266-5_9

[49] A. Ribas and J. D. Wolchok , “Cancer Immunotherapy Using Checkpoint Blockade,” Science 359, no. 6382 (2018): 1350–1355. 29567705 10.1126/science.aar4060 PMC7391259

[50] L. Meng , H. Wu , J. Wu , et al., “Mechanisms of Immune Checkpoint Inhibitors: Insights Into the Regulation of Circular RNAS Involved in Cancer Hallmarks,” Cell Death & Disease 15, no. 1 (2024): 3. 38177102 10.1038/s41419-023-06389-5 PMC10766988

[51] D. B. Doroshow , S. Bhalla , M. B. Beasley , et al., “PD‐L1 as a Biomarker of Response to Immune‐Checkpoint Inhibitors,” Nature Reviews. Clinical Oncology 18, no. 6 (2021): 345–362. 10.1038/s41571-021-00473-5 33580222

[52] Z. Duan , R. Shi , B. Gao , and J. Cai , “N‐Linked Glycosylation of PD‐L1/PD‐1: An Emerging Target for Cancer Diagnosis and Treatment,” Journal of Translational Medicine 22, no. 1 (2024): 705. 39080767 10.1186/s12967-024-05502-2 PMC11290144

[53] D. D. Shen , Y. P. Bi , J. R. Pang , et al., “Generation, Secretion and Degradation of Cancer Immunotherapy Target PD‐L1,” Cellular and Molecular Life Sciences 79, no. 8 (2022): 413. 35819633 10.1007/s00018-022-04431-x PMC11073444

[54] Y. Gao , N. T. Nihira , X. Bu , et al., “Acetylation‐Dependent Regulation of PD‐L1 Nuclear Translocation Dictates the Efficacy of Anti‐PD‐1 Immunotherapy,” Nature Cell Biology 22, no. 9 (2020): 1064–1075. 32839551 10.1038/s41556-020-0562-4 PMC7484128

[55] C. E. Murray , A. V. R. Kornepati , C. Ontiveros , et al., “Tumour‐Intrinsic PDL1 Signals Regulate the Chk2 DNA Damage Response in Cancer Cells and Mediate Resistance to Chk1 Inhibitors,” Molecular Cancer 23, no. 1 (2024): 242. 39478560 10.1186/s12943-024-02147-z PMC11523829

[56] Z. Wu , Z. Cao , H. Yao , et al., “Coupled Deglycosylation‐Ubiquitination Cascade in Regulating PD‐1 Degradation by MDM2,” Cell Reports 42, no. 7 (2023): 112693. 37379210 10.1016/j.celrep.2023.112693

[57] R. Wang , S. He , J. Long , et al., “Emerging Therapeutic Frontiers in Cancer: Insights Into Posttranslational Modifications of PD‐1/PD‐L1 and Regulatory Pathways,” Experimental Hematology & Oncology 13, no. 1 (2024): 46. 38654302 10.1186/s40164-024-00515-5 PMC11040904

[58] Q. Gou , C. Dong , H. Xu , et al., “PD‐L1 Degradation Pathway and Immunotherapy for Cancer,” Cell Death & Disease 11, no. 11 (2020): 955. 33159034 10.1038/s41419-020-03140-2 PMC7648632

[59] W. Zhang , D. Li , X. Xu , et al., “A Bispecific Peptide‐Polymer Conjugate Bridging Target‐Effector Cells to Enhance Immunotherapy,” Advanced Healthcare Materials 12, no. 18 (2023): e2202977. 36878223 10.1002/adhm.202202977

[60] A. Piermattei , G. Migliara , A. Feraco , et al., “Evaluation of PD‐L1, TERT Promoter Mutations, and BRAFV600E Mutation in Poorly Differentiated, Differentiated High Grade Thyroid Carcinoma and Anaplastic Carcinoma of the Thyroid: Our Institutional Experience,” Virchows Archiv 487 (2025): 605–618. 40481961 10.1007/s00428-025-04134-1

[61] L. Shobab , D. Al‐Souri , L. Mathews‐Kim , et al., “PD‐L1 Expression Varies in Thyroid Cancer Types and Is Associated With Decreased Progression Free Survival (PFS) in Patients With Anaplastic Thyroid Cancer,” Cancers (Basel) 16, no. 21 (2024): 3632. 39518072 10.3390/cancers16213632 PMC11545090

[62] N. Anand , P. Srivastava , N. Husain , D. Agarwal , A. Gupta , and R. Pradhan , “Evaluation of CTLA‐4 and PD‐L1 Expression in Thyroid Carcinoma and Its Prognostic Significance,” Cureus 16, no. 8 (2024): e67004. 39286684 10.7759/cureus.67004 PMC11403645

[63] M. Sajedi Shacker , A. R. Dehghanian , R. Kiani , M. R. Haghshenas , and N. Erfani , “High Expression of Immune Checkpoint Molecules in Different Types of Thyroid Cancer,” Iranian Journal of Allergy, Asthma, and Immunology 23, no. 5 (2024): 514–525. 39586745 10.18502/ijaai.v23i5.16747

[64] X. Zhu , C. Hu , Z. Zhang , et al., “PD‐L1 and B7‐H3 Are Effective Prognostic Factors and Potential Therapeutic Targets for High‐Risk Thyroid Cancer,” Endocrine Pathology 35, no. 3 (2024): 230–244. 39102163 10.1007/s12022-024-09822-3

[65] S. Agarwal , C. K. Jung , P. Gaddam , et al., “PD‐L1 Expression and Its Modulating Factors in Anaplastic Thyroid Carcinoma: A Multi‐Institutional Study,” American Journal of Surgical Pathology 48, no. 10 (2024): 1233–1244. 39004795 10.1097/PAS.0000000000002284

[66] M. Boruah , P. Gaddam , S. Agarwal , et al., “PD‐L1 Expression in Rare and Aggressive Thyroid Cancers: A Preliminary Investigation for a Role of Immunotherapy,” Journal of Cancer Research and Therapeutics 19, no. 2 (2023): 312–320. 37006068 10.4103/jcrt.jcrt_1471_22

[67] Y. Luo , Y. C. Yang , C. K. Shen , et al., “Immune Checkpoint Protein Expression Defines the Prognosis of Advanced Thyroid Carcinoma,” Frontiers in Endocrinology (Lausanne) 13 (2022): 859013. 10.3389/fendo.2022.859013 PMC9094437 35574031

[68] P. Adam , S. Kircher , I. Sbiera , et al., “FGF‐Receptors and PD‐L1 in Anaplastic and Poorly Differentiated Thyroid Cancer: Evaluation of the Preclinical Rationale,” Frontiers in Endocrinology (Lausanne) 12 (2021): 712107. 10.3389/fendo.2021.712107 PMC8406771 34475850

[69] J. Lin , Y. Qiu , X. Zheng , Y. Dai , and T. Xu , “The miR‐199a‐5p/PD‐L1 Axis Regulates Cell Proliferation, Migration and Invasion in Follicular Thyroid Carcinoma,” BMC Cancer 22, no. 1 (2022): 756. 35818041 10.1186/s12885-022-09838-0 PMC9275143

[70] V. B. Santana , V. M. Krüger , M. C. Y. Abrahão , et al., “Chronic Lymphocytic Thyroiditis With Oncocytic Metaplasia Influences PD‐L1 Expression in Papillary Thyroid Carcinoma,” Head and Neck Pathology 18, no. 1 (2024): 14. 38457034 10.1007/s12105-024-01618-5 PMC10923758

[71] L. Bernadett , K. Alexandra , F. Georgina , et al., “No Correlation Between PD‐L1 and NIS Expression in Lymph Node Metastatic Papillary Thyroid Carcinoma,” Diagnostics (Basel, Switzerland) 14, no. 17 (2024): 1858. 39272644 10.3390/diagnostics14171858 PMC11394040

[72] A. K. Siraj , S. K. Parvathareddy , P. Pratheeshkumar , et al., “PD‐L1 Is an Independent Prognostic Marker in Middle Eastern PTC and Its Expression Is Upregulated by BRAFV600E Mutation,” Cancers (Basel) 13, no. 3 (2021): 555. 33535609 10.3390/cancers13030555 PMC7867170

[73] B. Kovacevic , D. Vucevic , S. Cerovic , and C. Eloy , “Peripheral Versus Intraparenchymal Papillary Thyroid Microcarcinoma: Different Morphologies and PD‐L1 Expression,” Head and Neck Pathology 16, no. 1 (2022): 200–212. 34076845 10.1007/s12105-021-01337-1 PMC9018942

[74] D. Wusiman , L. Guo , L. Li , et al., “Clinicopathological and Prognostic Significance of PD‐L1 and TIM‐3 Expression in Medullary Thyroid Carcinoma: A Retrospective Immunohistochemistry Study,” Journal of Endocrinological Investigation 47, no. 1 (2024): 91–100. 37464189 10.1007/s40618-023-02126-z PMC10776706

[75] Y. Bai , T. Guo , D. Niu , et al., “Clinical Significance and Interrelations of PD‐L1 Expression, Ki‐67 Index, and Molecular Alterations in Sporadic Medullary Thyroid Carcinoma From a Chinese Population,” Virchows Archiv 481, no. 6 (2022): 903–911. 35920918 10.1007/s00428-022-03390-9

[76] A. S. Harahap , F. K. Lay , R. Kodariah , F. J. Wongkar , and M. F. Ham , “Association of Programmed Death‐Ligand 1 Expression With Aggressive Histological Types of Thyroid Carcinoma,” Cancer Management and Research 14 (2022): 3539–3550. 36583030 10.2147/CMAR.S392475 PMC9793731

[77] D. Y. Oh , A. Algazi , J. Capdevila , et al., “Efficacy and Safety of Pembrolizumab Monotherapy in Patients With Advanced Thyroid Cancer in the Phase 2 KEYNOTE‐158 Study,” Cancer 129, no. 8 (2023): 1195–1204. 36748723 10.1002/cncr.34657

[78] B. Chern , D. Pinto , J. H. Lum , and R. Parameswaran , “Nearly Half of Patients With Anaplastic Thyroid Cancer May be Amenable to Immunotherapy,” Biomedicine 12, no. 6 (2024): 1304. 10.3390/biomedicines12061304 PMC11201491 38927511

[79] C. Feng , Y. Tao , C. Yu , L. Wang , X. Liu , and Y. Cao , “Integrative Single‐Cell Transcriptome Analysis Reveals Immune Suppressive Landscape in the Anaplastic Thyroid Cancer,” Cancer Gene Therapy 30, no. 12 (2023): 1598–1609. 37679527 10.1038/s41417-023-00663-6

[80] S. K. Yoo , Y. S. Song , E. K. Lee , et al., “Integrative Analysis of Genomic and Transcriptomic Characteristics Associated With Progression of Aggressive Thyroid Cancer,” Nature Communications 10, no. 1 (2019): 2764. 10.1038/s41467-019-10680-5 PMC6591357 31235699

[81] N. Agrawal , Y. Jiao , M. Sausen , et al., “Exomic Sequencing of Medullary Thyroid Cancer Reveals Dominant and Mutually Exclusive Oncogenic Mutations in RET and RAS,” Journal of Clinical Endocrinology and Metabolism 98, no. 2 (2013): E364–E369. 23264394 10.1210/jc.2012-2703 PMC3565108

[82] D. L. Jardim , A. Goodman , D. de Melo Gagliato , and R. Kurzrock , “The Challenges of Tumor Mutational Burden as an Immunotherapy Biomarker,” Cancer Cell 39, no. 2 (2021): 154–173. 33125859 10.1016/j.ccell.2020.10.001 PMC7878292

[83] J. Liu , Z. Chen , Y. Li , W. Zhao , J. Wu , and Z. Zhang , “PD‐1/PD‐L1 Checkpoint Inhibitors in Tumor Immunotherapy,” Frontiers in Pharmacology 12 (2021): 731798. 34539412 10.3389/fphar.2021.731798 PMC8440961

[84] K. Aliazis , A. Christofides , R. Shah , et al., “The Tumor Microenvironment's Role in the Response to Immune Checkpoint Blockade,” Nature Cancer 6, no. 6 (2025): 924–937. 40514448 10.1038/s43018-025-00986-3 PMC12317369

[85] A. C. Huang , M. A. Postow , R. J. Orlowski , et al., “T‐Cell Invigoration to Tumour Burden Ratio Associated With Anti‐PD‐1 Response,” Nature 545, no. 7652 (2017): 60–65. 28397821 10.1038/nature22079 PMC5554367

[86] L. Chen , X. Jiang , Y. Li , et al., “How to Overcome Tumor Resistance to Anti‐PD‐1/PD‐L1 Therapy by Immunotherapy Modifying the Tumor Microenvironment in MSS CRC,” Clinical Immunology 237 (2022): 108962. 35227870 10.1016/j.clim.2022.108962

[87] B. C. Bertol , E. S. Bales , J. D. Calhoun , et al., “Lenvatinib Plus Anti‐PD‐1 Combination Therapy for Advanced Cancers: Defining Mechanisms of Resistance in an Inducible Transgenic Model of Thyroid Cancer,” Thyroid 32, no. 2 (2022): 153–163. 34641722 10.1089/thy.2021.0371 PMC8861922

[88] L. P. Andrews , H. Yano , and D. A. A. Vignali , “Inhibitory Receptors and Ligands Beyond PD‐1, PD‐L1 and CTLA‐4: Breakthroughs or Backups,” Nature Immunology 20, no. 11 (2019): 1425–1434. 31611702 10.1038/s41590-019-0512-0

[89] G. Varricchi , S. Loffredo , G. Marone , et al., “The Immune Landscape of Thyroid Cancer in the Context of Immune Checkpoint Inhibition,” International Journal of Molecular Sciences 20, no. 16 (2019): 3934. 31412566 10.3390/ijms20163934 PMC6720642

[90] A. Strati , C. Adamopoulos , I. Kotsantis , A. Psyrri , E. Lianidou , and A. G. Papavassiliou , “Targeting the PD‐1/PD‐L1 Signaling Pathway for Cancer Therapy: Focus on Biomarkers,” International Journal of Molecular Sciences 26, no. 3 (2025): 1235. 39941003 10.3390/ijms26031235 PMC11818137

[91] H. Zhang , L. Gong , L. Yu , et al., “Emerging Roles of Non‐Coding RNA Derived From Extracellular Vesicles in Regulating PD‐1/PD‐L1 Pathway: Insights Into Cancer Immunotherapy and Clinical Applications,” Cancer Cell International 25, no. 1 (2025): 188. 40410719 10.1186/s12935-025-03809-8 PMC12103061

[92] S. Gettinger , J. Choi , K. Hastings , et al., “Impaired HLA Class I Antigen Processing and Presentation as a Mechanism of Acquired Resistance to Immune Checkpoint Inhibitors in Lung Cancer,” Cancer Discovery 7, no. 12 (2017): 1420–1435. 29025772 10.1158/2159-8290.CD-17-0593 PMC5718941

[93] M. D. Vesely , T. Zhang , and L. Chen , “Resistance Mechanisms to Anti‐PD Cancer Immunotherapy,” Annual Review of Immunology 40 (2022): 45–74. 10.1146/annurev-immunol-070621-030155 35471840

[94] S. Yang , W. Wei , and Q. Zhao , “B7‐H3, a Checkpoint Molecule, as a Target for Cancer Immunotherapy,” International Journal of Biological Sciences 16, no. 11 (2020): 1767–1773. 32398947 10.7150/ijbs.41105 PMC7211166

[95] K. Yonesaka , K. Haratani , S. Takamura , et al., “B7‐H3 Negatively Modulates CTL‐Mediated Cancer Immunity,” Clinical Cancer Research 24, no. 11 (2018): 2653–2664. 29530936 10.1158/1078-0432.CCR-17-2852

[96] W. T. Zhou and W. L. Jin , “B7‐H3/CD276: An Emerging Cancer Immunotherapy,” Frontiers in Immunology 12 (2021): 701006. 34349762 10.3389/fimmu.2021.701006 PMC8326801

[97] B. Zhao , H. Li , Y. Xia , et al., “Immune Checkpoint of B7‐H3 in Cancer: From Immunology to Clinical Immunotherapy,” Journal of Hematology & Oncology 15, no. 1 (2022): 153. 36284349 10.1186/s13045-022-01364-7 PMC9597993

[98] K. Hińcza‐Nowak , A. Kowalik , A. Walczyk , et al., “CD276 as a Candidate Target for Immunotherapy in Medullary Thyroid Cancer,” International Journal of Molecular Sciences 24, no. 12 (2023): 10019. 37373167 10.3390/ijms241210019 PMC10298428

[99] B. Zhao , Z. Huang , X. Zhu , et al., “Clinical Significance of the Expression of co‐Stimulatory Molecule B7‐H3 in Papillary Thyroid Carcinoma,” Frontiers in Cell and Development Biology 10 (2022): 819236. 10.3389/fcell.2022.819236 PMC9039293 35493085

[100] N. Sobhani , D. R. Tardiel‐Cyril , A. Davtyan , D. Generali , R. Roudi , and Y. Li , “CTLA‐4 in Regulatory T Cells for Cancer Immunotherapy,” Cancers (Basel) 13, no. 6 (2021): 1440. 33809974 10.3390/cancers13061440 PMC8005092

[101] E. I. Buchbinder and A. Desai , “CTLA‐4 and PD‐1 Pathways: Similarities, Differences, and Implications of Their Inhibition,” American Journal of Clinical Oncology 39, no. 1 (2016): 98–106. 26558876 10.1097/COC.0000000000000239 PMC4892769

[102] X. Shi , C. W. Li , L. C. Tan , et al., “Immune co‐Inhibitory Receptors PD‐1, CTLA‐4, TIM‐3, LAG‐3, and TIGIT in Medullary Thyroid Cancers: A Large Cohort Study,” Journal of Clinical Endocrinology and Metabolism 106, no. 1 (2021): 120–132. 33000173 10.1210/clinem/dgaa701

[103] P. Song , Y. Xu , and G. Ye , “B7‐H3 and ICAM‐1 Are Potentially Therapeutic Targets for Thyroid Carcinoma,” Diagnostic Pathology 19, no. 1 (2024): 77. 38858715 10.1186/s13000-024-01504-2 PMC11163747

[104] P. Gu , B. Ling , W. Ma , et al., “Indoleamine 2,3‐Dioxygenase 2 Immunohistochemical Expression in Medullary Thyroid Carcinoma: Implications in Prognosis and Immunomodulatory Effects,” BMC Cancer 22, no. 1 (2022): 1116. 36319978 10.1186/s12885-022-10173-7 PMC9624013

[105] X. Zheng , R. Sun , and T. Wei , “Immune Microenvironment in Papillary Thyroid Carcinoma: Roles of Immune Cells and Checkpoints in Disease Progression and Therapeutic Implications,” Frontiers in Immunology 15 (2024): 1438235. 39290709 10.3389/fimmu.2024.1438235 PMC11405226

[106] Y. Ming , D. Gongye , and B. Min , “Dysfunction of Natural Killer Cells Mediated by PD‐1 and Tim‐3 Pathway in Anaplastic Thyroid Cancer,” International Immunopharmacology 64 (2018): 333–339. 30243069 10.1016/j.intimp.2018.09.016

[107] Z. Wu , S. Li , and X. Zhu , “The Mechanism of Stimulating and Mobilizing the Immune System Enhancing the Anti‐Tumor Immunity,” Frontiers in Immunology 12 (2021): 682435. 34194437 10.3389/fimmu.2021.682435 PMC8237941

[108] P. Song , G. Pan , Y. Zhang , et al., “Prospects and Challenges of Immunotherapy for Thyroid Cancer,” Endocrine Practice 31 (2025): 373–379. 39631664 10.1016/j.eprac.2024.11.012

[109] S. Iwama , T. Kobayashi , and H. Arima , “Management, Biomarkers and Prognosis in People Developing Endocrinopathies Associated With Immune Checkpoint Inhibitors,” Nature Reviews. Endocrinology 21 (2025): 289–300. 10.1038/s41574-024-01077-6 39779950

[110] T. Mosaferi , K. Tsai , S. Sovich , et al., “Optimal Thyroid Hormone Replacement Dose in Immune Checkpoint Inhibitor‐Associated Hypothyroidism Is Distinct From Hashimoto's Thyroiditis,” Thyroid 32, no. 5 (2022): 496–504. 35199588 10.1089/thy.2021.0685 PMC9145255

[111] K. Wang , Y. Zhang , Y. Xing , H. Wang , M. He , and R. Guo , “Current and Future of Immunotherapy for Thyroid Cancer Based on Bibliometrics and Clinical Trials,” Discover Oncology 15, no. 1 (2024): 50. 38403820 10.1007/s12672-024-00904-6 PMC10894806

[112] R. K. Vaddepally , P. Kharel , R. Pandey , R. Garje , and A. B. Chandra , “Review of Indications of FDA‐Approved Immune Checkpoint Inhibitors Per NCCN Guidelines With the Level of Evidence,” Cancers (Basel) 12, no. 3 (2020): 738. 32245016 10.3390/cancers12030738 PMC7140028

[113] Y. Shiravand , F. Khodadadi , S. M. A. Kashani , et al., “Immune Checkpoint Inhibitors in Cancer Therapy,” Current Oncology (Toronto, Ont.) 29, no. 5 (2022): 3044–3060. 35621637 10.3390/curroncol29050247 PMC9139602

[114] J. M. Mehnert , A. Varga , M. S. Brose , et al., “Safety and Antitumor Activity of the Anti‐PD‐1 Antibody Pembrolizumab in Patients With Advanced, PD‐L1‐Positive Papillary or Follicular Thyroid Cancer,” BMC Cancer 19, no. 1 (2019): 196. 30832606 10.1186/s12885-019-5380-3 PMC6399859

[115] J. Capdevila , L. J. Wirth , T. Ernst , et al., “PD‐1 Blockade in Anaplastic Thyroid Carcinoma,” Journal of Clinical Oncology 38, no. 23 (2020): 2620–2627. 32364844 10.1200/JCO.19.02727 PMC7476256

[116] K. Sehgal , T. Pappa , K. Y. Shin , et al., “Dual Immune Checkpoint Inhibition in Patients With Aggressive Thyroid Carcinoma: A Phase 2 Nonrandomized Clinical Trial,” JAMA Oncology 10, no. 12 (2024): 1663–1671. 39446365 10.1001/jamaoncol.2024.4019 PMC11581533

[117] D. Ji , W. Shen , M. Kuang , et al., “A Phase II Study to Evaluate the Efficacy and Safety of Camrelizumab Plus Famitinib in Advanced or Metastatic Thyroid Cancer,” Journal of Clinical Oncology 40, no. 16_suppl (2022): 6085.

[118] J. D. French , B. R. Haugen , F. P. Worden , et al., “Combination Targeted Therapy With Pembrolizumab and Lenvatinib in Progressive, Radioiodine‐Refractory Differentiated Thyroid Cancers,” Clinical Cancer Research 30, no. 17 (2024): 3757–3767. 38922338 10.1158/1078-0432.CCR-23-3417 PMC11883846

[119] M. E. Cabanillas , R. Dadu , R. Ferrarotto , et al., “Anti‐Programmed Death Ligand 1 Plus Targeted Therapy in Anaplastic Thyroid Carcinoma: A Nonrandomized Clinical Trial,” JAMA Oncology 10, no. 12 (2024): 1672–1680. 39446377 10.1001/jamaoncol.2024.4729 PMC11581602

[120] A. Hatashima , B. Archambeau , H. Armbruster , et al., “An Evaluation of Clinical Efficacy of Immune Checkpoint Inhibitors for Patients With Anaplastic Thyroid Carcinoma,” Thyroid 32, no. 8 (2022): 926–936. 35583228 10.1089/thy.2022.0073

[121] S. Moog , L. Lamartina , M. A. Bani , et al., “Alkylating Agent‐Induced High Tumor Mutational Burden in Medullary Thyroid Cancer and Response to Immune Checkpoint Inhibitors: Two Case Reports,” Thyroid 33, no. 11 (2023): 1368–1373. 37698883 10.1089/thy.2023.0144

[122] J. Robert , S. Raghav , and S. J. L. Joline , “Immune Checkpoint Inhibitor Combinations‐Current and Emerging Strategies,” British Journal of Cancer 128, no. 8 (2023): 1415–1417. 36747017 10.1038/s41416-023-02181-6 PMC10070427

[123] V. Somayeh , O. Z. Angelina , K. Ramadhan Ado , et al., “Combination Therapy With Immune Checkpoint Inhibitors (ICIs); a New Frontier,” Cancer Cell International 22, no. 1 (2022): 2. 34980128 10.1186/s12935-021-02407-8 PMC8725311

[124] G. Wu , Y. Song , S. Yang , et al., “The Role of Targeted Therapy and/or Immunotherapy Therapy in Anaplastic Thyroid Carcinoma,” Endocrine 84, no. 3 (2024): 1013–1020. 38146047 10.1007/s12020-023-03647-6

[125] D. Soll , P. Bischoff , A. Frisch , et al., “First Effectiveness Data of Lenvatinib and Pembrolizumab as First‐Line Therapy in Advanced Anaplastic Thyroid Cancer: A Retrospective Cohort Study,” BMC Endocrine Disorders 24, no. 1 (2024): 25. 38383419 10.1186/s12902-024-01555-y PMC10882904

[126] J. Li , X. Zhang , Z. Mu , D. Sun , Y. Sun , and Y. Lin , “Response to Apatinib and Camrelizumab Combined Treatment in a Radioiodine Refractory Differentiated Thyroid Cancer Patient Resistant to Prior Anti‐Angiogenic Therapy: A Case Report and Literature Review,” Frontiers in Immunology 13 (2022): 943916. 36003403 10.3389/fimmu.2022.943916 PMC9393697

[127] D. X. Ma , X. P. Ding , C. Zhang , and P. Shi , “Combined Targeted Therapy and Immunotherapy in Anaplastic Thyroid Carcinoma With Distant Metastasis: A Case Report,” World Journal of Clinical Cases 10, no. 12 (2022): 3849–3855. 35647147 10.12998/wjcc.v10.i12.3849 PMC9100742

[128] Y. Song , Y. Zhang , Y. Bai , et al., “Combination Kinase Inhibitors and Immunotherapy for Unresectable Anaplastic Thyroid Carcinoma: A Retrospective Single‐Center Study,” Oral Oncology 159 (2024): 107067. 39395384 10.1016/j.oraloncology.2024.107067

[129] D. Barbaro , R. Forleo , M. A. Profilo , et al., “Neoadjuvant Treatment With Lenvatinib and Pembrolizumab in a BRAF V600E‐Mutated Anaplastic Thyroid Cancer: A Case Report,” Frontiers in Endocrinology (Lausanne) 15 (2024): 1389294. 10.3389/fendo.2024.1389294 PMC11263007 39045273

[130] S. Hamidi , R. Dadu , M. E. Zafereo , et al., “Initial Management of BRAF V600E‐Variant Anaplastic Thyroid Cancer: The FAST Multidisciplinary Group Consensus Statement,” JAMA Oncology 10, no. 9 (2024): 1264–1271. 38990526 10.1001/jamaoncol.2024.2133

[131] S. S. Du , G. W. Chen , P. Yang , et al., “Radiation Therapy Promotes Hepatocellular Carcinoma Immune Cloaking via PD‐L1 Upregulation Induced by cGAS‐STING Activation,” International Journal of Radiation Oncology, Biology, Physics 112, no. 5 (2022): 1243–1255. 34986380 10.1016/j.ijrobp.2021.12.162

[132] E. Chajon , J. Castelli , H. Marsiglia , and R. De Crevoisier , “The Synergistic Effect of Radiotherapy and Immunotherapy: A Promising but Not Simple Partnership,” Critical Reviews in Oncology/Hematology 111 (2017): 124–132. 28259287 10.1016/j.critrevonc.2017.01.017

[133] S. Wächter , S. Roth , N. Gercke , et al., “Anti‐Proliferative Effect of Radiotherapy and Implication of Immunotherapy in Anaplastic Thyroid Cancer Cells,” Life (Basel) 13, no. 6 (2023): 1397. 37374179 10.3390/life13061397 PMC10301015

[134] Y. Xing , Y. Wang , and X. Wu , “Radiotherapy Combined With Immunotherapy Successfully Treated One Case of Anaplastic Thyroid Cancer: A Case Report,” Frontiers in Oncology 13 (2023): 1125226. 37256174 10.3389/fonc.2023.1125226 PMC10225731

[135] D. Goh , K. H. Lim , S. R. B. Sudirman , M. K. Ang , M. L. K. Chua , and C. M. Lim , “Boosted Abscopal Effect From Radiotherapy and Pembrolizumab in Anaplastic Thyroid Cancer: A Mini‐Review and Case Report,” Chinese Clinical Oncology 12, no. 5 (2023): 57. 37964542 10.21037/cco-23-55

[136] N. Y. Lee , N. Riaz , V. Wu , et al., “A Pilot Study of Durvalumab (MEDI4736) With Tremelimumab in Combination With Image‐Guided Stereotactic Body Radiotherapy in the Treatment of Metastatic Anaplastic Thyroid Cancer,” Thyroid 32, no. 7 (2022): 799–806. 35521657 10.1089/thy.2022.0050 PMC9293682

[137] Z. Sun , C. Wang , Y. Zhao , and Q. Ling , “CAR‐T Cell Therapy in Advanced Thyroid Cancer: From Basic to Clinical,” Frontiers in Immunology 15 (2024): 1411300. 38911868 10.3389/fimmu.2024.1411300 PMC11190081

[138] T. Chen , M. Wang , Y. Chen , and Y. Liu , “Current Challenges and Therapeutic Advances of CAR‐T Cell Therapy for Solid Tumors,” Cancer Cell International 24, no. 1 (2024): 133. 38622705 10.1186/s12935-024-03315-3 PMC11017638

[139] H. Li , X. Zhou , G. Wang , et al., “CAR‐T Cells Targeting TSHR Demonstrate Safety and Potent Preclinical Activity Against Differentiated Thyroid Cancer,” Journal of Clinical Endocrinology and Metabolism 107, no. 4 (2022): 1110–1126. 34751400 10.1210/clinem/dgab819

[140] J. Ding , D. Li , X. Liu , et al., “Chimeric Antigen Receptor T‐Cell Therapy for Relapsed and Refractory Thyroid Cancer,” Experimental Hematology & Oncology 11, no. 1 (2022): 59. 36138444 10.1186/s40164-022-00311-z PMC9494903

[141] T. A. Erickson , Y. P. Shih , J. Fass , M. Jang , and E. Tran , “T Cells Engineered to Express Immunoreceptors Targeting the Frequently Expressed Medullary Thyroid Cancer Antigens Calcitonin, CEA, and RET M918T,” Thyroid 32, no. 7 (2022): 789–798. 35587601 10.1089/thy.2022.0020

[142] Y. Zhang , Z. Liu , W. Wei , and Y. Li , “TCR Engineered T Cells for Solid Tumor Immunotherapy,” Experimental Hematology & Oncology 11, no. 1 (2022): 38. 35725570 10.1186/s40164-022-00291-0 PMC9210724

[143] Y. Ping , C. Liu , and Y. Zhang , “T‐Cell Receptor‐Engineered T Cells for Cancer Treatment: Current Status and Future Directions,” Protein & Cell 9, no. 3 (2018): 254–266. 28108950 10.1007/s13238-016-0367-1 PMC5829268

[144] L. Cui , C. Zhang , H. Ding , et al., “Clonal Distribution and Intratumor Heterogeneity of the TCR Repertoire in Papillary Thyroid Cancer With or Without Coexistent Hashimoto's Thyroiditis,” Frontiers in Immunology 13 (2022): 821601. 35720279 10.3389/fimmu.2022.821601 PMC9203861

[145] J. Zhou , C. Zhang , W. Mao , et al., “Development of TSHR‐CAR NK‐92 Cells for Differentiated Thyroid Cancer,” Molecular and Cellular Endocrinology 589 (2024): 112251. 38670219 10.1016/j.mce.2024.112251

[146] S. Wang , J. Sun , K. Chen , et al., “Perspectives of Tumor‐Infiltrating Lymphocyte Treatment in Solid Tumors,” BMC Medicine 19, no. 1 (2021): 140. 34112147 10.1186/s12916-021-02006-4 PMC8194199

[147] M. Nam , W. Yang , H. S. Kim , et al., “Papillary Thyroid Cancer Immune Phenotypes via Tumor‐Infiltrating Lymphocyte Spatial Analysis,” Endocrine‐Related Cancer 30, no. 9 (2023): e230110. 37279258 10.1530/ERC-23-0110

[148] A. Betof Warner , P. G. Corrie , and O. Hamid , “Tumor‐Infiltrating Lymphocyte Therapy in Melanoma: Facts to the Future,” Clinical Cancer Research 29, no. 10 (2023): 1835–1854. 36485001 10.1158/1078-0432.CCR-22-1922 PMC10183807

[149] T. Matsutani , E. Akbay , and E. Elkord , “Editorial: Novel Biomarkers in Tumor Immunity and Immunotherapy,” Frontiers in Immunology 15 (2024): 1405082. 38650943 10.3389/fimmu.2024.1405082 PMC11033507

[150] C. Quan , O. Belaydi , J. Hu , et al., “N(6)‐Methyladenosine in Cancer Immunotherapy: An Undervalued Therapeutic Target,” Frontiers in Immunology 12 (2021): 697026. 34526985 10.3389/fimmu.2021.697026 PMC8436617

[151] J. He , M. Zhou , J. Yin , et al., “METTL3 Restrains Papillary Thyroid Cancer Progression via m(6)A/c‐Rel/IL‐8‐Mediated Neutrophil Infiltration,” Molecular Therapy 29, no. 5 (2021): 1821–1837. 33484966 10.1016/j.ymthe.2021.01.019 PMC8116572

[152] X. Ruan , M. Tian , N. Kang , et al., “Genome‐Wide Identification of m6A‐Associated Functional SNPs as Potential Functional Variants for Thyroid Cancer,” American Journal of Cancer Research 11, no. 11 (2021): 5402–5414. 34873468 PMC8640822

[153] W. Wang , Q. Huang , Z. Liao , et al., “ALKBH5 Prevents Hepatocellular Carcinoma Progression by Post‐Transcriptional Inhibition of PAQR4 in an m6A Dependent Manner,” Experimental Hematology & Oncology 12, no. 1 (2023): 1. 36609413 10.1186/s40164-022-00370-2 PMC9825045

[154] N. Kang , Z. Zhao , Z. Wang , et al., “METTL3 Regulates Thyroid Cancer Differentiation and Chemosensitivity by Modulating PAX8,” International Journal of Biological Sciences 20, no. 9 (2024): 3426–3441. 38993572 10.7150/ijbs.84797 PMC11234206

[155] F. H. Ji , Z. Yang , C. Sun , S. Lowe , and X. G. Qiu , “Characterization of m6A Methylation Modifications and Tumor Microenvironment Infiltration in Thyroid Cancer,” Clinical & Translational Oncology 25, no. 1 (2023): 269–282. 36163443 10.1007/s12094-022-02940-6

[156] W. Zhou , J. Lin , J. Liu , et al., “Thyroid Cancer Risk Prediction Model Using m6A RNA Methylation Regulators: Integrated Bioinformatics Analysis and Histological Validation,” Aging (Albany NY) 15, no. 3 (2023): 846–865. 36791151 10.18632/aging.204525 PMC9970309

[157] L. Cai , T. Liu , H. Hua , X. Jiang , and L. Qian , “m6A Modification Patterns Are Associated With Copy Number Burden and Tumor Immune Landscape in Thyroid Cancer,” BMC Endocrine Disorders 23, no. 1 (2023): 271. 38057752 10.1186/s12902-023-01510-3 PMC10699001

[158] M. Xia , S. Wang , Y. Ye , Y. Tu , T. Huang , and L. Gao , “Effect of the m6ARNA Gene on the Prognosis of Thyroid Cancer, Immune Infiltration, and Promising Immunotherapy,” Frontiers in Immunology 13 (2022): 995645. 36389678 10.3389/fimmu.2022.995645 PMC9664221

[159] Y. Su , B. Xu , J. Li , et al., “Identification of m6A‐Associated LncRNAs as Predict Factors for the Immune Infiltration and Prognosis of Thyroid Cancer,” Annals of Medicine 55, no. 1 (2023): 1298–1316. 36974635 10.1080/07853890.2023.2192049 PMC10054316

[160] D. Chen , H. Zhao , Z. Guo , et al., “Identification of m6A‐Related lncRNAs LINC02471 and DOCK9‐DT as Potential Biomarkers for Thyroid Cancer,” International Immunopharmacology 133 (2024): 112050. 38636370 10.1016/j.intimp.2024.112050

[161] H. Feng , J. Feng , X. Han , et al., “The Potential of Siglecs and Sialic Acids as Biomarkers and Therapeutic Targets in Tumor Immunotherapy,” Cancers (Basel) 16, no. 2 (2024): 289. 38254780 10.3390/cancers16020289 PMC10813689

[162] X. Hou , C. Chen , X. Lan , and X. He , “Unveiling the Molecular Features, Relevant Immune and Clinical Characteristics of SIGLEC15 in Thyroid Cancer,” Frontiers in Immunology 13 (2022): 975787. 36159823 10.3389/fimmu.2022.975787 PMC9500188

[163] L. Bao , Y. Li , X. Hu , et al., “Targeting SIGLEC15 as an Emerging Immunotherapy for Anaplastic Thyroid Cancer,” International Immunopharmacology 133 (2024): 112102. 38652971 10.1016/j.intimp.2024.112102

[164] T. Jin , W. Wang , L. Ge , X. Li , and M. Ge , “The Expression of Two Immunosuppressive SIGLEC Family Molecules in Papillary Thyroid Cancer and Their Effect on Prognosis,” Endocrine 82, no. 3 (2023): 590–601. 37480496 10.1007/s12020-023-03452-1

[165] Y. Zhang , J. Dai , T. Wu , N. Yang , and Z. Yin , “The Study of the Coexistence of Hashimoto's Thyroiditis With Papillary Thyroid Carcinoma,” Journal of Cancer Research and Clinical Oncology 140, no. 6 (2014): 1021–1026. 24619663 10.1007/s00432-014-1629-z PMC11823965

[166] C. de Resen Paiva , C. Grønhøj , U. Feldt‐Rasmussen , and C. von Buchwald , “Association Between Hashimoto's Thyroiditis and Thyroid Cancer in 64,628 Patients,” Frontiers in Oncology 7 (2017): 53. 28443243 10.3389/fonc.2017.00053 PMC5385456

[167] S. Xu , H. Huang , J. Qian , et al., “Prevalence of Hashimoto Thyroiditis in Adults With Papillary Thyroid Cancer and Its Association With Cancer Recurrence and Outcomes,” JAMA Network Open 4, no. 7 (2021): e2118526. 34313737 10.1001/jamanetworkopen.2021.18526 PMC8317012

[168] S. K. Park , J. H. Ryoo , M. H. Kim , et al., “Association Between Eight Autoimmune Diseases and Thyroid Cancer: A Nationwide Cohort Study,” Thyroid 34, no. 2 (2024): 206–214. 38149584 10.1089/thy.2023.0353

[169] Y. K. Chen , C. L. Lin , F. T. Cheng , F. C. Sung , and C. H. Kao , “Cancer Risk in Patients With Hashimoto's Thyroiditis: A Nationwide Cohort Study,” British Journal of Cancer 109, no. 9 (2013): 2496–2501. 24084773 10.1038/bjc.2013.597 PMC3817335

[170] Y. Li , Y. Zang , T. Fan , et al., “Transcriptomic Signatures Associated With Autoimmune Thyroiditis in Papillary Thyroid Carcinoma and Cancer Immunotherapy‐Induced Thyroid Dysfunction,” Computational and Structural Biotechnology Journal 20 (2022): 2391–2401. 35664236 10.1016/j.csbj.2022.05.019 PMC9125670

[171] H. Ma , G. Li , D. Huo , et al., “Impact of Hashimoto's Thyroiditis on the Tumor Microenvironment in Papillary Thyroid Cancer: Insights From Single‐Cell Analysis,” Frontiers in Endocrinology (Lausanne) 15 (2024): 1339473. 10.3389/fendo.2024.1339473 PMC11439672 39351536

[172] F. Pani , Y. Yasuda , S. T. Rousseau , et al., “Preconditioning of the Immune System Modulates the Response of Papillary Thyroid Cancer to Immune Checkpoint Inhibitors,” Journal for Immunotherapy of Cancer 10, no. 12 (2022): e005538. 36521928 10.1136/jitc-2022-005538 PMC9756278

[173] F. Pani , P. Caria , Y. Yasuda , et al., “The Immune Landscape of Papillary Thyroid Cancer in the Context of Autoimmune Thyroiditis,” Cancers 14, no. 17 (2022): 4287. 36077831 10.3390/cancers14174287 PMC9454449

[174] G. Laetitia , S. Sven , and J. Fabrice , “Combinatorial Therapies in Thyroid Cancer: An Overview of Preclinical and Clinical Progresses,” Cells 9, no. 4 (2020): 830. 32235612 10.3390/cells9040830 PMC7226736

[175] S. M. Park , C. J. Chen , D. J. Verdon , et al., “Proliferating Macrophages in Human Tumours Show Characteristics of Monocytes Responding to Myelopoietic Growth Factors,” Frontiers in Immunology 15 (2024): 1412076. 38903497 10.3389/fimmu.2024.1412076 PMC11188303

